# A proposed molecular mechanism for pathogenesis of severe RNA-viral pulmonary infections

**DOI:** 10.12688/f1000research.25390.2

**Published:** 2021-01-06

**Authors:** Peter K. Rogan, Eliseos J. Mucaki, Ben C. Shirley

**Affiliations:** 1 Biochemistry, University of Western Ontario, London, Ontario, N6A 2C8, Canada; 2 CytoGnomix Inc, London, Ontario, N5X 3X5, Canada

**Keywords:** SARS-CoV-2, Influenza A, HIV-1, Dengue Virus, Apoptosis, R-loop, DNA damage, RNA binding protein

## Abstract

**Background:** Certain riboviruses can cause severe pulmonary complications leading to death in some infected patients. We propose that DNA damage induced-apoptosis accelerates viral release, triggered by depletion of host RNA binding proteins (RBPs) from nuclear RNA bound to replicating viral sequences.

**Methods:** Information theory-based analysis of interactions between RBPs and individual sequences in the Severe Acute Respiratory Syndrome CoronaVirus 2 (SARS-CoV-2), Influenza A (H3N2), HIV-1, and Dengue genomes identifies strong RBP binding sites in these viral genomes. Replication and expression of viral sequences is expected to increasingly sequester RBPs - SRSF1 and RNPS1. Ordinarily, RBPs bound to nascent host transcripts prevents their annealing to complementary DNA. Their depletion induces destabilizing R-loops. Chromosomal breakage occurs when an excess of unresolved R-loops collide with incoming replication forks, overwhelming the DNA repair machinery. We estimated stoichiometry of inhibition of RBPs in host nuclear RNA by counting competing binding sites in replicating viral genomes and host RNA.

**Results:** Host RBP binding sites are frequent and conserved among different strains of RNA viral genomes. Similar binding motifs of SRSF1 and RNPS1 explain why DNA damage resulting from SRSF1 depletion is complemented by expression of RNPS1. Clustering of strong RBP binding sites coincides with the distribution of RNA-DNA hybridization sites across the genome. SARS-CoV-2 replication is estimated to require 32.5-41.8 hours to effectively compete for binding of an equal proportion of SRSF1 binding sites in host encoded nuclear RNAs. Significant changes in expression of transcripts encoding DNA repair and apoptotic proteins were found in an analysis of influenza A and Dengue-infected cells in some individuals.

**Conclusions:** R-loop-induced apoptosis indirectly resulting from viral replication could release significant quantities of membrane-associated virions into neighboring alveoli. These could infect adjacent pneumocytes and other tissues, rapidly compromising lung function, causing multiorgan system failure and other described symptoms.

## Introduction

### Background

RNA viruses have long been known as an important source of zoonotic disease transmission ^1^. In these infections, a key question that needs to be answered is which infected individuals will progress from mild to severe symptoms that require intensive care? While complex underlying conditions increase susceptibility, severe acute respiratory syndrome coronavirus 2 (SARS-CoV-2) and Influenza A can lead to severe or lethal outcomes regardless of the age or health status in certain individuals. The Chinese and the initial US patients with SARS-CoV-2 showed that higher viral replication and multiplicity of infection are evident in severely ill individuals ^2– 4^. Textbook depictions of viral release and infection indicate budding from the cell membrane. This explanation might not adequately explain the rapid onset of symptoms and transmissibility seen in some individuals infected with these agents. We suggest that these factors can be explained by a cytopathology of induced lytic events, releasing high titers of virus. Programmed cell death (apoptosis), which has been suggested to occur in RNA viral conditions such as Influenza, is activated through innate immunity, with concomitant inflammatory responses. Viral RNA has been suggested to signal Toll-Like receptors and type I interferon expression, which binds to its receptor, IFNAR, and stimulates induction of PCD genes such as FasL or TRAIL ^5^.

**Figure 1.**
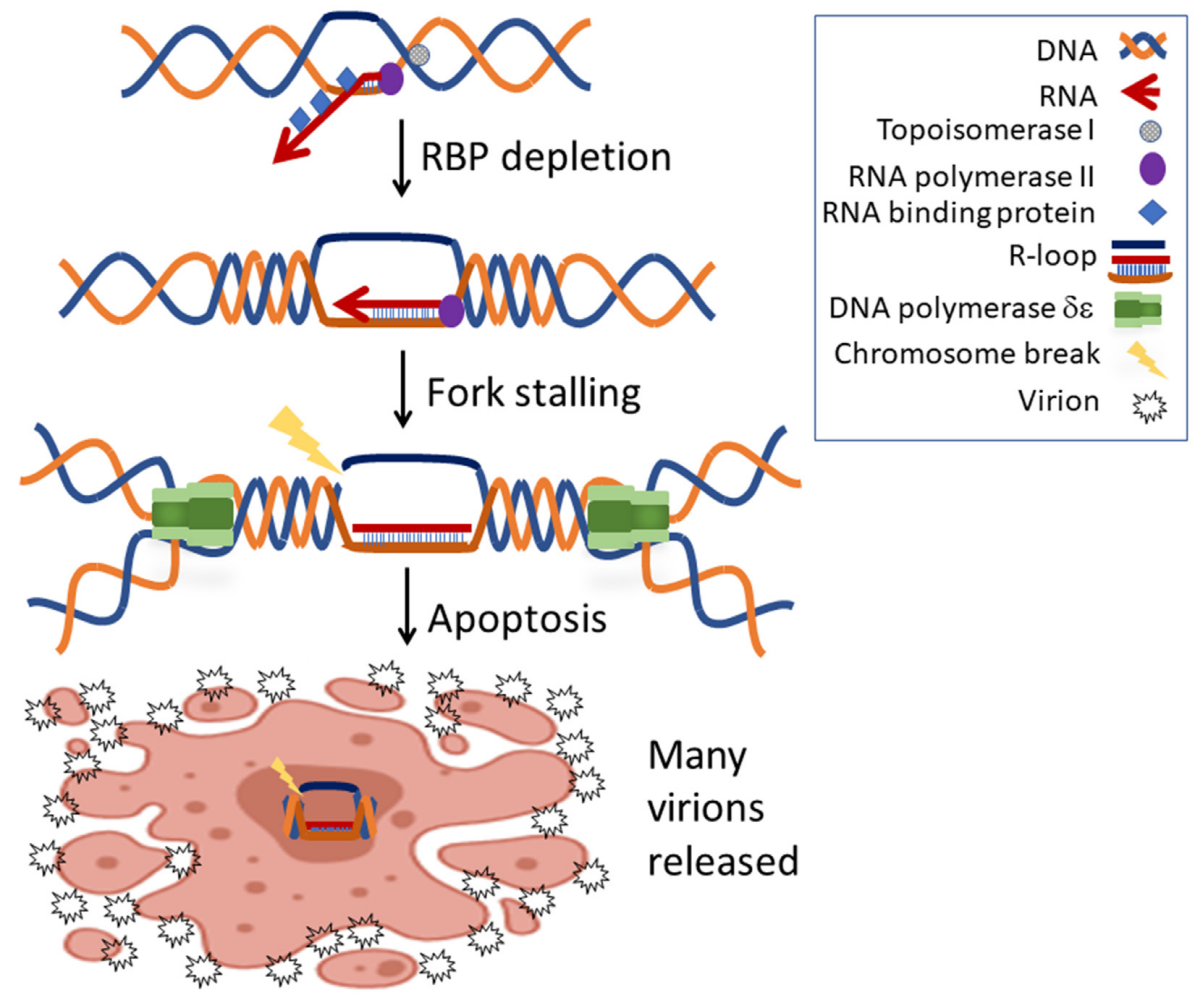
Proposed mechanism of high multiplicity of RNA viral infections. Newly synthesized host RNA binding proteins (SRSF1, RNPS1) are required to stabilize nascent transcripts throughout the nucleus. During influenza or other viral infections, these proteins can be bound to viral genomes and transcriptomes. As viral replication and transcription proceeds, these nucleic acids containing strong binding sites for these RBPs in the cytoplasm (SARS-CoV-2) and nucleus (Influenza) that complete with host RNAs and deplete these proteins from the nucleus. This enables nascent transcripts to reanneal with transcription templates, and R-loops are formed. If not removed by RNAse H or other helicases, unresolved R-loops at numerous genomic loci triggers genomic instability. Their frequency and density of unrepaired chromosome damage would be expected to overwhelm DNA repair components (BRCA1/2, FANC complex, and XPC), inducing multiple chromosomal strand breaks in each cell ^10^. These breakage events initiate apoptosis, releasing a high multiplicity of infectious viral particles.

We propose an alternative mechanism in which infection of RNA virus triggers unrepaired sites of chromosomal breakage, causing apoptosis and consequentially, high-titer viral release ( Figure 1). This is precipitated by the binding of RNA binding proteins (RBPs) to viral genomes and transcripts instead of nuclear transcripts, to prevent destabilization of chromosome structure. This study identifies the sequences, locations and abundance of these binding sites and presents evidence for specific expression changes in DNA damage genes in Influenza and Dengue infections and evidence of expression changes consistent with induction of apoptosis. The damage is thought to arise as the result of replication forks colliding with R-loops formed by host transcripts. Ordinarily these structures are mitigated through formation of stable interactions with frequently bound endogenous RBPs ^6^.

The SR protein family consists of RNA binding proteins that play significant roles in the regulation of mRNA splicing ^7^. SRSF1 (formerly ASF/SF2) is an exonic splicing enhancer (ESE) that has been shown to interact with the U1 snRNP and recruit the protein to the donor (5’) splice site ^8, 9^. However, binding of SRSF1 to nascent transcripts has also been shown to play a significant role in genome stability, first described in reference 10, whereby the presence of SRSF1 bound to pre-mRNA repressed the formation of DNA:RNA hybrids, which led to R-loops, double-stranded breaks, and a hypermutation phenotype. This phenotype could be corrected not only by increasing RNase H expression (to eliminate DNA:RNA hybrids), but with the overexpression of the RNA binding protein RNPS1 ^11^. RNPS1, part of the apoptosis-and splicing-associated protein (ASAP) complex, can directly interact with SRSF1 ^12^ and could possibly help recruit SRSF1 to ESE sites ^13^. Other RNA binding proteins have been shown to increase genome instability when depleted, including *THOC1* ^14^, *MFAP1* ^15^, and *FIP1L1* ^16^.

Binding sites for these RBPs are identified using information theory (IT)-based sequence analysis, which has proven both theoretically and in numerous practical examples to be an accurate approach for predicting binding affinities of nucleic acid sequences recognized by particular DNA or RNA binding proteins ^17^. IT can be used to identify binding sites, and to evaluate the impact a sequence variant may have on binding site strength ^18^. IT has been applied in studies which involved mRNA splicing ^19, 20^, splicing regulatory factors (SRFs ^21, 22^), other RNA binding proteins ^23^ and transcription factor binding sites (TFBS ^24, 25^), and has been used to accurately predicted level of gene expression and identify causative mutations in a wide spectrum of diseases ^17^. IT-based analysis has the distinct advantage to other bioinformatic approaches as the predicted information content (known as *R _i_*; measured in bits) can be quantified as binding site affinity as it is related to thermodynamic entropy ^26^. The binding affinity of a sequence predicted by IT has been shown experimentally to directly relate to the observed binding quantity of said sequence ^26^. IT-based models are generated from a series of annotated binding sites for a particular RBP. The average strength of the sites used to generate said is referred to as its *R _sequence_*. IT-based models can also be derived from high-throughput binding site identification techniques such as ChIP-seq (e.g. the derivation of TFBS models in 24). Information density-based clustering (IDBC) analysis, where groups of closely situated binding sites are evaluated based on their combined strength (their “information density”) and intersite distances, has been applied along with these TFBS models in both the identification of TFBS-dense clusters, and accurate prediction of gene expression patterns ^25^.

We and others have suggested that the viral genome binds to these RBPs (e.g. SRSF1 is enriched among SARS-CoV-2 RNA-protein interactions ^27^) as well, and we define the locations of likely strong binding sites across the genomes of various RNA viruses. We propose that the replicating viral genome and transcriptome binds and sequesters these proteins, preventing their reimportation into the nucleus where they are normally needed for essential post-transcriptional activities. We theorize that incremental replication and transcription of viral RNAs in the cytoplasm creates a sink for these proteins, starving the host nucleus, and initiating a series of events that release viral particles into the lumen, enabling rapid infection of neighboring lung epithelial cells ( Figure 1). An infographic has been created to provide a detailed step-by-step guide to the proposed mechanism, from the initial viral infection to spread of infection to the lungs and other major organs, leading to lowered blood oxygen levels, and multi-system organ failure ^28^.

### Proposed molecular pathogenetic mechanism of RNA-viral infection

RNA viral genomes of Influenza viruses replicate in the nucleus and are processed by host RNA spliceosomes. For example, the M and NS segments of the Influenza genome are processed using the host splicing mechanism ^29^. Viral RNAs, like host transcripts, are capable of sequence specific binding to RBPs. This can conceivably deplete RBPs from host encoded RNAs, where they ordinarily function. These unbound RNAs are capable of hybridizing to the non-template derived strand of the chromosome ^30^. RNA naturally forms a stronger bond to DNA than DNA does to itself, especially rG:dC hybrids ^10^. As a result, mRNAs would replace DNA by hybridizing complimentary bases, resulting in R-loop formation, and can lead to DNA damage.

The RNA spliceosome regulator SRSF1 acts on exonic splicing enhancer sequences in pre-mRNA and forms RNP complexes with nascent mRNA precursors. Aside from its established role in enhancing exon recognition ^9^, binding of SRSF1 to these transcripts is required to prevent or destabilize the formation of R-loops ^10^. R-loops are derived from RNA transcripts that anneal to the chromosomal strand complimentary to the transcription template stand. If not eliminated, these structures pose a threat to genomic integrity as targets for DNA damage. The structure of R-loops consists of two duplex-single strand junctions which are recognized by nucleases that cleave the DNA ^30^. DNA fragmentation causes a G2 phase cell cycle arrest which can potentially lead to cell death ^11^. R-loops that are not targeted by nucleases are nonetheless still non-functional and thus, inflict damage on the cell ^10^. As RNA viruses enter the cell and replicate, the nucleic acid sequences they encode divert RBPs such as SRSF1 away from binding to nuclear RNA transcripts, thus promoting the creation of R-loops.

RNPS1 is a pre-mRNA splicing activator protein that functions together with SRSF1 to form RNP complexes on nascent transcripts ^13, 31^, but also has a role in preventing transcriptional R-loop formation ^11^. RNPS1 also suppresses high molecular weight DNA fragmentation at high expression levels. These two proteins work together but have independent mechanisms as RNPS1 cannot compensate for SRSF1 splicing function in its absence and vice versa ^11^.

In Dengue virus, the protein called NS5 binds to host spliceosome complexes and modulates endogenous splicing to change mRNA isoform abundance of antiviral factors. By also interacting with U5 snRNP particles, it reduces the efficiency of pre-mRNA processing, hence resulting in a less restrictive environment for viral replication. It has also been shown that NS5 interacts with the host protein, RNPS1, which disrupts normal nuclear RNA binding processes ^32^.

Viral infections interfere with post-transcriptional processing of host pre-mRNA including splicing, capping, and translation during viral invasion. Since SRSF1 binds and interacts with pre-mRNA during the earliest stages of splicing, diversion of SRSF1 and other spliceosomes to other RNA sequences depletes the cell’s resources. Normally, cellular mRNA is 7-methylguanosine cap is added to the 5’ end to protect the sequence from degradation. However, Influenza carries proteins that has “cap-snatching” abilities ^33^. Influenza snatches the 5’ cap by cleaving the mRNA 10 to 15 nucleotides away from the guanosine and this cap is used to prime transcription of the virus. Finally, during viral infections, all RNA processing mechanisms are now being shared between two genomes. Ultimately, as transcriptional and translation mechanisms fail to facilitate the mRNA, they will create R-loops with DNA, cause DNA damage, and induce higher expression of DNA repair genes (such as *DDB2;* see Results).

Unrepaired damaged DNA that encounters a replication fork leads to unresolved double strand breaks, triggering apoptosis. The quantity of virus that escapes into tissues, blood and other conduits (e.g. lymphatic), and other systems would likely dwarf the amount that is released by conventional viral budding from the cell membrane. This viral load will likely overwhelm the immune system in individuals who are already immune deficient and might provoke a systemic inflammatory response (like a cytokine storm). However, the high titer of virus is likely to infect neighboring cells and other tissues. The extent of the apoptotic response may be the distinguishing finding which separates the patients who survive the infection from those who end up in intensive care, develop pulmonary insufficiency and multi-system failure.

The deficiency in SRSF1 and other RBPs in the nuclei of Influenza, Dengue or SARS-CoV-2 infected cells does not require any specialized mechanism. Assuming that the virus is replicating freely in the cytoplasm (or nucleus, in the case of Influenza), the significant excess of unpackaged, replicated viral RNA acts as a sponge to sequester newly synthesized, folded RBPs. Based on mass action, the quantity of RBPs that would be transported into the nucleus for host mRNA processing would have a much-diminished nuclear stoichiometry in comparison with normal, uninfected cells.

## Results

### Derivation of CLIP-based SRSF1 and RNPS1 information theory-based models

Cells depleted of SRSF1 has been shown to have unstable genomes which can be corrected by overexpression of RNPS1 ^11^. In order to investigate the significance of SRSF1 and RNPS1 binding in viral genomes, we first developed information theory-based models for the recognition sequences for each of these proteins using binding site datasets derived from transcriptome-wide RNA binding protein datasets of CLIP sequencing data. We then scanned multiple RNA viral genomes, as well as the human transcriptome, with these derived models to identify and predict the strength of individual binding sites.

An Information Weight Matrix (IWM) for SRSF1 has been previously derived ^21^, however, it was only based on very small set of manually curated binding sites (N=28). We therefore derived new SRSF1 IWMs using publicly available eCLIP data (two separate replicates from 34). Multiple SRSF1 models exhibited very similar binding motifs, however, their differences justified our analyses using the two most divergent IWMs in this study. These models are referred to as SRSF1 “Replicate 1” and “Replicate 2” models, as they are models derived from two separate eCLIP experimental replicates from the same study. SRSF1 “Replicate 1” is derived from a larger number of eCLIP peaks (50,000) compared to 5,000 for “Replicate 2”. Since SRSF1 “Replicate 1” was derived from a greater number of sites, it therefore may be more accurate for detection of weaker SRSF1 binding sites.

A distinct IWM was derived by iCLIP data from transcriptome-wide, protein crosslinking to sequences recognized by RNPS1 ^31^. It was evident that the RNPS1 IWM and the newly derived SRSF1 models exhibited a similar pattern of nucleotide conservation based on comparison of their respective sequence logos ( Table 1). STAMP, a software program which analyzes position weight matrices of nucleic acid (or protein) motifs, was used to compare these models based on their e-values ^35^. The SRSF1 “Replicate 1” and “Replicate 2” models were both highly similar (motif alignment e-value < 0.01) to the RNPS1 IWM ( Table 1), implying that individual binding sites recognized by these two factors are similar. Indeed, the motif similarity between these two factors has been described ^13^. We suggest that this overlap in their respective binding affinities may account for why RNPS1 overexpression can enable SRSF1-deficient cells to overcome their inherent genomic instability phenotype.

**Table 1.** Comparison of Derived SRSF1 and RNPS1 Information Models and Binding Sites in Genomes.

Factor		SRSF1 [Rep1] ^1, 2^		SRSF1 [Rep1] / RNPS1 Model Comparison		RNPS1 ^1^		SRSF1 [Rep2] / RNPS1 Model Comparison		SRSF1 [Rep2] ^1, 2^	
Sequence Logo		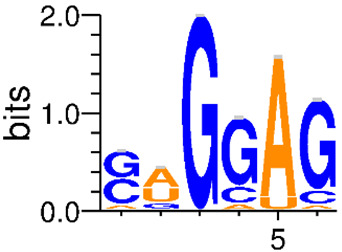		-		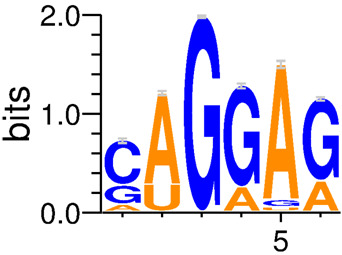		-		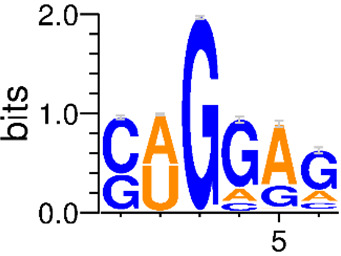	
*R _sequence_* (bits)		6.7 ± 2.1		-		7.8 ± 1.9		-		6.4 ± 2.1	
Motif Similarity (E-value) ^3^		-		5.0e-09		-		1.1e-09		-	
No. of Expressed Binding Sites (A549; ≥ 0 bits) ^4^		1.3e08		5.4e07 (57%) ^7^		9.3e07		6.4e07 (69%)		1.5e08	
No. of Expressed Binding Sites (Pneumocytes; ≥0 bits) ^6^		6.8e07		2.9e07 (58%)		5.0e07		3.4e07 (69%)		7.9e07	
No. of Sites (SARS-CoV-2; +|- strand)	≥ 0 bits	860	732	435 (72%)	305 (65%)	608	466	363 (60%)	273 (59%)	810	772
	*≥* 1/2 *Rseq*	311	232	131 (51%)	86 (44%)	256	196	155 (61%)	115 (59%)	376	358
	*≥ Rseq*	31	42	16 (46%)	10 (40%)	35	25	35 (100%)	25 (100%)	60	33
No. of Sites (Influenza A; +|- strand) ^6^	≥ 0 bits	697	339	289 (61%)	118 (63%)	475	188	268 (56%)	129 (69%)	616	388
	*≥* 1/2 *Rseq*	263	118	122 (49%)	47 (55%)	248	85	162 (65%)	65 (76%)	373	188
	*≥ Rseq*	50	23	24 (53%)	12 (75%)	45	16	45 (100%)	16 (100%)	84	35

^1^ RNPS1 model derived from publicly available iCLIP data (E-MTAB-4215; ArrayExpress), while SRSF1 models were derived from eCLIP data (ENCSR456FVU; ENCODE Data Coordination Center); ^2^ SRSF1 [Rep1] and [Rep2] were derived from eCLIP dataset replicate 1 [50,000 peaks] and replicate 2 [5,000 peaks], respectively; ^3^ RNA binding motifs were compared using STAMP ^35^ using the Pearson Correlation Coefficient distance metric ^36^; ^4^ A549 cell line expression from GSE141171 dataset; ^5^ Primary type II pneumocyte expression from GSE86618 dataset; ^6^ Influenza A virus H3N2 strain (Ontario/104-25/2012). ^7^ RNPS1 sites used as denominator for all percentages.

We also derived IWMs from negative controls in the eCLIP and iCLIP resources which consisted of sequence libraries constructed from crosslinking studies of mock substrates ^31, 34^. The resultant IWMs did not resemble those obtained from crosslinking RNPS1 and SRSF1 to their cognate binding sites. The LOD scores (logarithm of the odds ratio of the respective e-values of RBP motifs relative to different mock sequence motifs) ranged from 3.8 to 6.1 for RNPS1 and from 4.4 to 8.8 for SRSF1.

### RBP binding sites in RNA viral genomes

The newly derived SRSF1 and RNPS1 models (as well as an hnRNP A1 model to act as a positive control [its derivation described in 22], as the RBP has been shown to regulate transcription of beta coronaviral genes ^37^) were used to scan the genomes of multiple RNA viruses: Dengue (Type 3), HIV (Strain B and C), Influenza A (H3N2; two separate strains), and SARS-CoV-2 (NC_045512.2). In coronaviruses, the infectious particle contains the positive strand, but the negative strand copy of the RNA is generated for protein translation ^38^ and may be available to bind RBPs. Therefore, both the positive and negative strands of the viral genomes were scanned for SRSF1, RNPS1 and hnRNP A1 binding, regardless of the replication mechanism of the virus.

**Figure 2.**
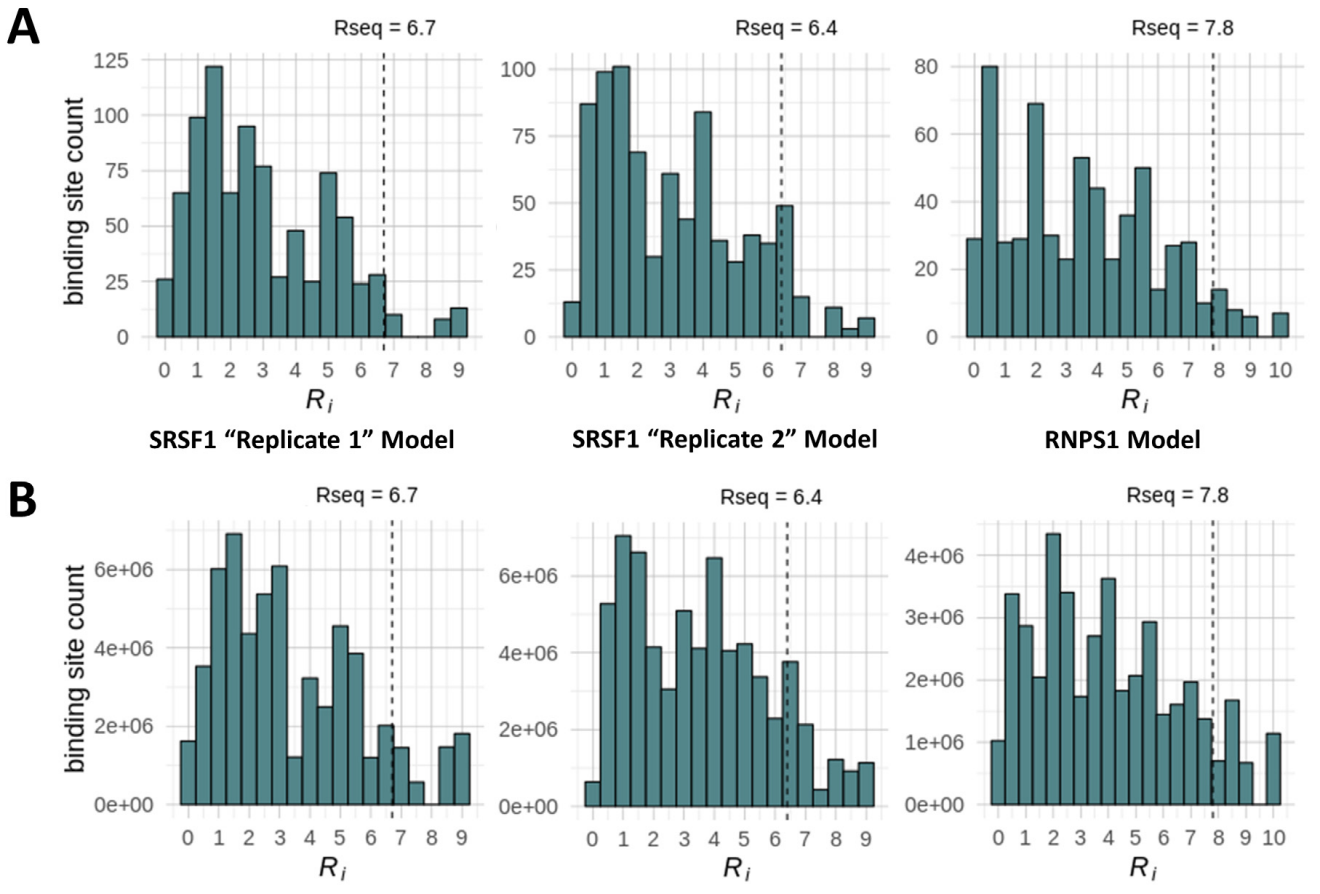
*R _i_* of SRSF1 and RNPS1 Binding Sites in the SARS-CoV-2 and Human Genomes. Histograms display the distribution of *R _i_* values for SRSF1 [“Replicate 1” and “Replicate 2” models] and RNPS1 binding sites strengths identified in **A**) the SARS-CoV-2 viral genome, and **B**) all transcribed regions in the human genome.

The SARS-CoV-2 genome was determined to contain >600 SRSF1 (with either SRSF1 model) and RNPS1 binding sites ( Table 1). However, histograms which illustrate the distribution of the strengths of all SRSF1 and RNPS1 binding sites in SARS-CoV-2 ( Figure 2A) reveal that the majority of these are weak sites (where *R _i_* < *R _sequence_*) that may not be used. We therefore focused downstream analysis on strong binding sites (where *R _i_* ≥ *R _sequence_*) of each IWM ( *R _sequence_*: 6.7 bits for the SRSF1 “Replicate 1” model; 6.4 bits for the SRSF1 “Replicate 2” model; 7.8 bits for the RNPS1 model; and 4.6 bits for the hnRNP A1 model). There are only 35 RNPS1 and between 31-60 SRSF1 binding sites (depending on SRSF1 model) on the positive strand of the SARS-CoV-2 genome that meet this *R _sequence_* threshold ( Table 1). The total number of SRSF1 binding sites within all other viral genomes tested are provided in Table 2, while RNPS1 and hnRNP A1 binding site counts are available within a Zenodo repository for this study (extended data ^39^ Section 1 – Table 1). The hnRNP A1 model consistently predicts more strong binding sites than the SRSF1 and RNPS1 models across all the RNA viral genomes tested, as well as in the human gene controls. This is likely partially due to its relatively low *R _sequence_* threshold compared to the other models used. Interestingly, we observed significantly more SRSF1 and RNPS1 binding sites on the positive strand compared to the negative strand for all tested RNA viral genomes (exception: sites in SARS-CoV-2 predicted by SRSF1 “Replicate 1” model). This phenomenon was observed in both positive-strand and negative-strand RNA viruses (e.g. both Influenza A strains tested). This imbalance was not observed in the human genes tested ( Table 2).

**Table 2.** SRSF1 Binding Site and Information-Dense Cluster Counts in RNA Viral Genomes.

Virus (Strain)	Length (nt)	SRSF1 Sites (Replicate 1; ≥Rseq [6.7 bits])			SRSF1 Clusters (Replicate 1; ≥0.1 bits)			Strongest SRSF1 Cluster (bits; Replicate 1)	SRSF1 Sites (Replicate 2; ≥Rseq [6.4 bits])			SRSF1 Clusters (Replicate 2; ≥0.1 bits)			Strongest SRSF1 Cluster (bits; Replicate 2)
		Both	+	-	Both	+	-		Both	+	-	Both	+	-	
**Dengue Virus** **(Type 3)**	10,707	65	47	18	28	26	2	162.1	107	85	22	26	24	2	107.1
**HIV-1 (Strain B)**	9,719	55	44	11	20	20	0	152.2	106	87	19	24	23	1	115.7
**HIV-1 (Strain C)**	9,031	58	48	10	21	18	3	142.8	103	81	22	16	14	2	143.8
**Influenza A** **(Ontario)**	13,151	73	50	23	23	21	2	100.4	119	84	35	24	21	3	111.9
**Influenza A** **(Shanghai)**	11,863	79	63	16	25	24	1	165.9	121	91	30	32	30	2	127.3
**SARS-CoV-2** **(NC_045512.2)**	30,899	73	31	42	10	5	5	69.9	93	60	33	7	5	2	66.7
***IKBKB*** **(Human Gene)**	61,352	615	305	310	244	124	120	288.2	803	410	393	288	143	145	577.1
***SIRT1*** **(Human Gene)**	33,721	225	109	116	72	30	42	526.0	283	125	158	80	37	43	152.6
***WDR4*** **(Human Gene)**	30,358	351	176	175	129	64	65	266.3	519	265	254	176	88	88	324.4

Columns labeled as “Both” indicate the number of binding sites or clusters on both strands of the viral genome.

Previously, tightly organized groups of transcription factor binding sites (TFBS) were identified using information dense clustering ^25, 40^. We applied this method to identify regions of the viral genomes with large concentrations of binding sites (extended data ^39^ Section 1 – Table 2). Clusters of weak SRSF1 and RNPS1 sites are common (e.g. there are 5 SRSF1 clusters on the positive strand of SARS-CoV-2; extended data ^39^ Section 1 – Tables 2A and 2B); however, clusters made up exclusively of strong binding sites ( *R _i_* ≥ *R _sequence_*) are extremely rare in the viral genomes tested.

**Figure 3.**
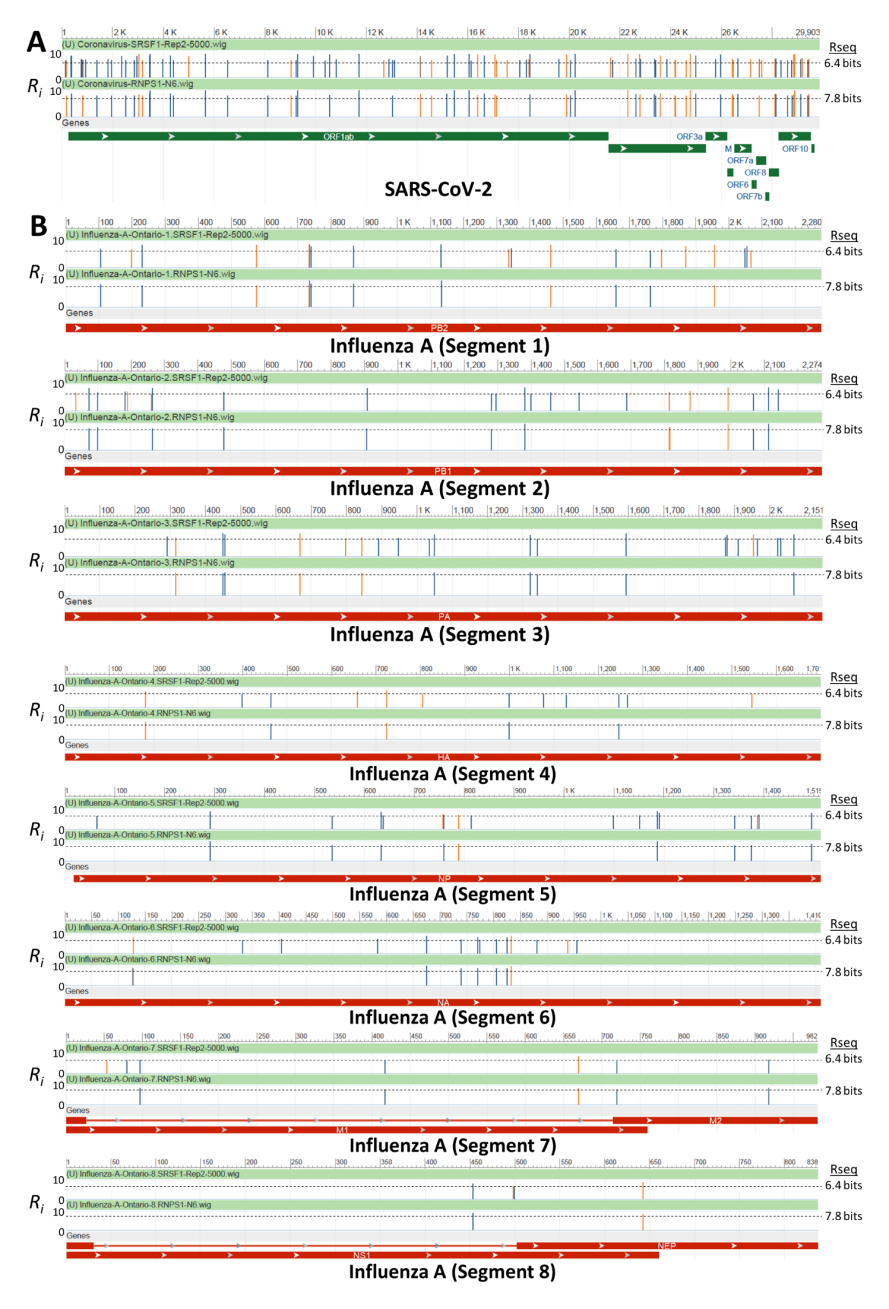
Distribution of SRSF1 and RNPS1 Binding Sites Across SARS-CoV-2 and Influenza A. The viral genomes of **A**) SARS-CoV-2 (NCBI Reference Sequence: NC_045512.2) and **B**) Influenza A virus (A/swine/Ontario/104-25/2012[H3N2]) were scanned for strong pre-existing binding sites for the RBP RNPS1 and SRSF1 (newly derived “Replicate 2” model). Custom wiggle tracks which contained those RBP of *R _i_* ≥ *R _sequence_* were generated and visualized by NCBI Nucleotide. Track images were manually adjusted to indicate the strand in which the binding site was identified (blue vertical lines indicate sites on the positive strand, orange on the negative strand). The majority of sites predicted by the RNPS1 model were simultaneously predicted by the SRSF1 model, however the SRSF1 model identifies additional unique binding sites.

We observed that all strong RNPS1 sites were also predicted to be strong ( *R _i_* ≥ *R _sequence_*) by the SRSF1 “Replicate 2” model. This is not surprising, as the two models were found to have significantly similar binding motifs ( Table 1). This overlap, as well as the location and strength of all other strong SRSF1 (“Replicate 2” model only) and RNPS1 binding sites, can be observed in Figure 3 where sites were mapped across the SARS-CoV-2 and Influenza A genomes. This was not observed, however, for SRSF1 “Replicate 1” despite its similarity to the RNPS1 model. For this SRSF1 model, nearly half of all strong RNPS1 sites were predicted to be weak ( *R _i_* below the *R _sequence_* threshold).

Despite its low mutation rate, over 220 SARS-CoV-2 strains have already been identified, with potential mutational hot spots of different geographic origins ^41^. If the proposed mechanism does play a role in the severity of infection, then it is expected that various strains of SARS-CoV-2 would not significantly differ in numbers of binding sites, as no particular strain of SARS-CoV-2 has yet been proven to affect disease recovery (indeed, more transmissible strains have been identified but none more pathogenic ^42, 43^). To test this theory, genomes of 8 SARS-CoV-2 strains were downloaded from the Global Initiative on Sharing All Influenza Data (GISAID) database and analyzed using the IWMs for SRSF1, RNPS1 and hnRNP A1 ( Table 3 for positive strand analysis; extended data ^39^ Section 1 – Table 3 for analysis of both strands). The particular strains that were selected were those that showed maximum divergence from one other based on analyses by NextStrain (which tracks the genomic epidemiology of SARS-CoV-2 ^44^). Binding site counts of different strains were within 90% across all strains, except for MT198652.1 (Spain), which contains an undetermined sequence where binding site differences are mapped. A strong consistency between binding site counts and strengths was noted, despite maximizing in the divergence between the selected SARS-CoV-2 strains. For RBPs binding, it was therefore not significant as to which SARS-CoV-2 sequence was selected for the subsequent analyses.

**Table 3.** Binding Site Counts in Genome Sequences of Multiple Coronavirus Strains (Positive Strand only).

	Model			
Coronavirus Strain	SRSF1 (Replicate 1)	SRSF1 (Replicate 2)	RNPS1	hnRNP A1
MT007544.1 (Australia)	31	60	35	573
MT066176.1 (Taiwan)	31	60	35	573
MT121215.1 (China)	31	60	35	573
MT163718.1 (USA)	31	60	35	573
MT188339.1 (USA)	31	60	35	572
MT198652.1 (Spain) ^a^	28	57	33	532
MT198653.1 (Spain) ^a^	31	60	35	558
NC_045512.2 (China)	31	60	35	573

^a^ Sequences contains a small stretch of undefined nucleotides, which is likely contributing to the lower number of binding sites found.

The absence of severe symptoms associated with the SARS-CoV-2 Singaporean strain (which features a deletion in ORF8 [pos. 27,848 to 28,229] ^45^), however, is not related to a significant loss of strong SRSF1 and RNPS1 binding sites. The SRSF1 “Replicate 1” model does not identify any binding sites (≥ *R _sequence_*) in this region. The SRSF1 “Replicate 2” model predicts 2 strong binding sites in this region, as does the RNPS1 model. There are 17 hnRNP A1 binding sites in this region, however there are 1,168 sites in total across the coronavirus genome; therefore, the missing hnRNP A1 sites account for only 1.4% of the total detectable hnRNP A1 binding sites.

Given the high Influenza A mutation rate, we evaluated the variability in RBP site count and affinities between strains, that is, whether these binding sites might be under selection for conservation of RBP binding. Four Influenza A strains (H3N2) from four separate clades (analogous to the SARS-CoV-2 strain selection procedure using NextStrain ^44^; A/Denmark/316/2020; A/England/323/2019; A/Singapore/TT0333/2019; and A/Sydney/1017/2018) along with the two Influenza A strains previously selected (A/swine/Ontario/104-25/2012 and A/Duck/Shanghai/C84/2009) were analyzed and their genomes scanned for the presence of strong RNPS1, SRSF1 and hnRNP A1 binding sites (extended data ^39^ Section 1 – Table 4). Depending on the strain, 13 to 16 RNPS1 and 30 to 35 SRSF1 (“Replicate 2” model) binding sites were identified on the negative strand of Influenza A (a range of 16–23 binding sites for SRSF1 “Replicate 1”, and 221 to 241 strong hnRNP A1 binding sites). Thus, it appears as though the overall number of binding sites remains relatively consistent between each Influenza A strain, despite their divergent genomic sequences.

The locations of all predicted binding sites and information-dense clusters within the genome of each RNA virus tested has been made available within the extended data archive (Section 2 ^39^). This data is provided in the form of ‘bedgraph’ genome browser tracks. The locations of binding site clusters are also provided as lollipop plots within the archive (Section 3), as are the IWMs used to evaluate each site (Section 4).

### Human transcriptome analysis of RNA binding sites

Each of these RNA viral genomes contain multiple strong RNA binding sites. The frequency of RBP binding in human transcriptomes was determined to relate the relative abundance of these proteins bound to viral RNAs compared to their normal reservoir in host nuclear RNA of infected cells. Expressed host gene sequences were scanned with IWMs for SRSF1, RNPS1 and hnRNP A1 to locate all potential binding sites throughout transcribed regions of the human genome, then partitioned among these genes based on their abundance in relevant cell types. These were compared with binding sites within 300nt of a known exon, as many of these RBPs have critical functions in exon recognition and maturation of mRNA splice isoforms (provided as bedgraph tracks in the Zenodo archive [Section 2] ^39^). While the majority of these binding sites are considered weak ( *R _i_* < *R _sequence_*; Figure 2B), the numbers of strong (binding sites with *R _i_* > *R _sequence_*) residing within transcribed regions are substantial (SRSF1 “Replicate 1” Model: 5,543,429; SRSF1 “Replicate 2” Model: 8,275,472; RNPS1: 4,368,943; hnRNP A1: 44,885,381). The intersite distance (the average distance between binding sites) appears to be inversely related to the overall number of binding sites, as the mean intersite distance between strong hnRNP A1 binding sites was considerably shorter than the distance between strong SRSF1 and RNPS1 binding sites (hnRNP A1: 24±40 nt; RNPS1: 149±248 nt; SRSF1 [“Replicate 1” model]: 105±241 nt; SRSF1 [“Replicate 2” model]: 89±197 nt; analysis using a maximum intersite distance threshold of 1,000nt). Regardless of these differences, however, this analysis illustrates that many strong binding sites are separated by < 200nt and highlights how densely arrayed these sites are in the human transcriptome.

**Figure 4.**
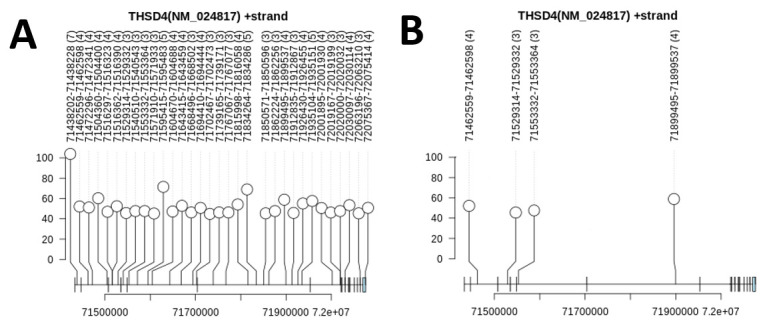
SRSF1 information dense clusters in the *THSD4* gene. **A**) Lollipop plot of information density of clusters annotated by coordinate range and number of sites comprising that cluster using the SRSF1 “Replicate 2” information-based weight models (all *R _i_* ≥ *R _sequence_*) for the NM_024817 mRNA splice form of *THSD4* (some clusters counted in Section 1 – Table 5 (extended data ^39^) are found in other *THSD4* splice forms which span beyond the range of this particular mRNA). **B**) Information dense SRSF1 clusters within *THSD4* that overlap a DRIPc-seq interval (GSE70189 DRIPc-seq dataset). One additional overlapping cluster is not displayed, as is located immediately upstream of the 5’ untranslated region of the NM_024817.2 splice form. No intervals from the GSE68845 DRIP-seq dataset overlap this gene.

The number of strong SRSF1, RNPS1 and hnRNP A1 binding sites ( *R _i_* ≥ *R _sequence_*) were enumerated by gene (extended data ^39^ Section 1 – Table 5 [A–D]; genes without any strong binding sites are not listed). Similar tables were created which count the number of information-dense clusters located within each gene (extended data ^39^ Section 1 - Table 5 [E–H]). In general, there were more hnRNP A1 clusters identified than SRSF1 and RNPS1 clusters (SRSF1 “Replicate 1” Model: 112,955; SRSF1 “Replicate 2” Model: 98,872; RNPS1: 39,285; hnRNP A1: 709,226), which is likely due to the higher frequency of strong hnRNP A1 sites and significantly lower hnRNP A1 intersite distance. Table 5 (from extended data ^39^ Section 1) also provides type II pneumocytes (from single-cell [sc] RNAseq data) and the A549 (human alveolar adenocarcinoma) cell line (RNAseq) expression values for each gene listed (in Transcripts Per Million [TPM]). Genes that are both highly expressed in lung cells and contain a high frequency of SRSF1 and/or RNPS1 information-dense binding site clusters would be considered strong candidate genes for R-loop formation in cells infected by an RNA virus. The gene *PTPRN2* has the highest total number of SRSF1 clusters (N=116 to 138 depending on the SRSF1 model used) but has relatively low level expression in pneumocytes (TPM = 0.052). The *THSD4* gene, however, has 35-36 high-density SRSF1 clusters (N=2,236-3,475 individual strong SRSF1 binding sites) and is expressed (≥ 1 TPM) in both lung cell expression data sets tested ( Figure 4A; extended data ^39^ Section 1 - Table 5 [E and F]). Overall, there are 1,225 genes with ≥10 SRSF1 and 127 genes with ≥10 RNPS1 information-dense clusters which are also expressed (TPM ≥ 1) in the expression datasets tested.

DRIP (DNA-RNA immunoprecipitation) sequencing is a high-throughput method of identifying regions of the genome where R-loops can form. DRIPc sequencing is an improvement which provides higher resolution mapping data in a strand-specific manner ^46^. To determine to what degree these DRIP-seq (GSE68845 [IMR90 cells]) and DRIPc-seq intervals (GSE70189 [NTERA2 cells]) overlapped RNPS1 and SRSF1 binding sites in uninfected cells, we performed an intersection between the two datasets and information dense clusters (extended data ^39^ Section 1 – Table 6 [A and B]) or individual binding sites (extended data ^39^ Section 1 – Table 6 [C and D]). It was uncommon for strong binding site clusters to overlap a DRIP-seq interval (0.4 – 1.7% of all transcriptome-wide clusters overlap a DRIP-seq interval). Despite an additional level of filtering (where the strand of the clusters and DRIPc-seq intervals must match), the frequency of overlap between binding site clusters and DRIPc-seq was much higher compared to the frequency of overlap to the DRIP-seq dataset (~15-17% overlap depending on IWM; extended data ^39^ Section 1 – Table 6A). In all test cases, limiting analysis to only those genes that are expressed in A549 cells (≥1 TPM) increased the percent overlap of clusters and both DRIP- and DRIPc-seq data sets (e.g. we find a 15.3% of RNPS1 clusters/DRIPc-seq overlap among all genes, but 20.2% overlap when considering expressed genes in the A549 cell line only). When this analysis was repeated but limited to only those clusters near an exon (within 300nt), this also showed a significant increase in the fraction of clusters overlapping DRIP-seq intervals (extended data ^39^ Section 1 – Table 6B). These observations remain consistent when considering individual binding sites, rather than binding site clusters (extended data ^39^ Section 1 – Table 6C and 6D). It therefore seems that the vast majority of individual binding sites and information-dense binding site clusters do not overlap these DRIP- and DRIPc-seq regions. For example, only 5 of 36 clusters within *THSD4* overlap the DRIPc-seq dataset ( Figure 4B; extended data ^39^ Section 1 – Table 5F).

Interestingly, the computed intersite distances for RNPS1, SRSF1 and hnRNP A1 binding sites that overlap DRIPc-seq intervals were shorter compared to the intersite distances of sites across the entire transcriptome (mean intersite distances: hnRNP A1: 22±45nt; RNPS1: 120±228nt; SRSF1 [“Replicate 1” model]: 76±205nt; SRSF1 [“Replicate 2” model]: 69±170nt; maximum intersite distance of 1,000nt). The general distributions of intersite distances between these two analyses were also found to be quite similar (extended data ^39^ Section 5). As we are limiting this analysis to sites that are within a few, often short DRIPc-seq intervals, the distances between pairs of sites are likely to be tightly grouped. We also computed the average number of all binding sites and clusters, and only those which overlap the DRIPc-seq dataset, for each individual gene (sites and clusters per 100nt of gene length; extended data ^39^ Section 1 - Table 5). Binding site densities within specific genes are reduced for sites overlapping DRIPc-seq intervals (e.g. *THSD4* SRSF1 cluster density reduces from 5.2E-03 to 7.0E-04 clusters per 100nt).

### DNA damage response by RNA viral infection

We have previously described a machine learning (ML) based approach for developing gene signatures for expression various environmental exposures to cells, initially focusing on prediction of chemotherapy effects ^47^. This method was applied to ionizing radiation data, from which accurate gene signatures were derived that could differentiate levels of radiation exposures. In particular, low exposures were distinguished from higher radiation levels that cause Acute Radiation Syndrome (ARS ^48^). ARS is characterized by vomiting, diarrhea, fever, low white blood cell count and fatigue. Physicians might not consider ARS in the differential diagnosis when presented with a patient exhibiting these symptoms, since Influenza and Dengue (viral) infections also present with vomiting, diarrhea, lymphopenia (especially Influenza H1N1 ^49^) and fatigue, and are more common. Like ARS, these conditions lead to death in some cases. While Influenza A has a worldwide distribution, Dengue is more prevalent in Southeast Asia, the Americas and the Western Pacific where it presents typically with severe manifestations including hemorrhagic fever and shock. We have considered how the life cycle of these viruses might be related to the corresponding cellular responses. 

Expression data from irradiated blood samples were used to derive the human radiation gene signatures reported in Zhao *et al.* ^48^. While it was assumed that these ML models were specific for diagnosing ARS, the models were further tested to determine if they could distinguish ARS from other conditions that share similar clinical presentation (e.g. vomiting, diarrhea). Four human ML radiation signatures from Zhao *et al.* (assessed by traditional validation; denotated as ML models “M1”, “M2”, “M3” and M4” which are described in extended data ^39^ Section 1 – Table 7) were used to evaluate 11 gene expression studies of patients infected with: Influenza (N=5, includes Influenza A [H3N2], swine flu [H1N1] and Influenza B viral infection data sets), Dengue virus (N=4) and aplastic anemia (N=2). On average, the ML models misclassified 26.4% of Influenza and 22.4% of Dengue patients as irradiated (Section 1 – Table 7). Approximately 15% of aplastic anemia patients were also misclassified. The model “M1” showed the lowest misclassification rate against Influenza patients (9–29% of patients misclassified), models “M2” best classified Dengue-infected patients (7–33% misclassified), while models “M1” and “M3” performed well with patients with aplastic anemia (5–20% misclassified for “M1” and 0–14% misclassified for “M3”). In nearly every instance, the inclusion of normal controls from the Influenza and Dengue studies improved overall accuracy of all four ML models (17.4% and 18.1% average misclassification of Influenza and Dengue-infected patients, respectively). This phenomenon was not observed in the aplastic anemia dataset tested. The observation that normal controls are more often correctly classified indicates that these models are not so much incorrectly classifying infected patients, as they are identifying gene expression differences that may be a response to or caused by the viral infection itself.

**Figure 5.**
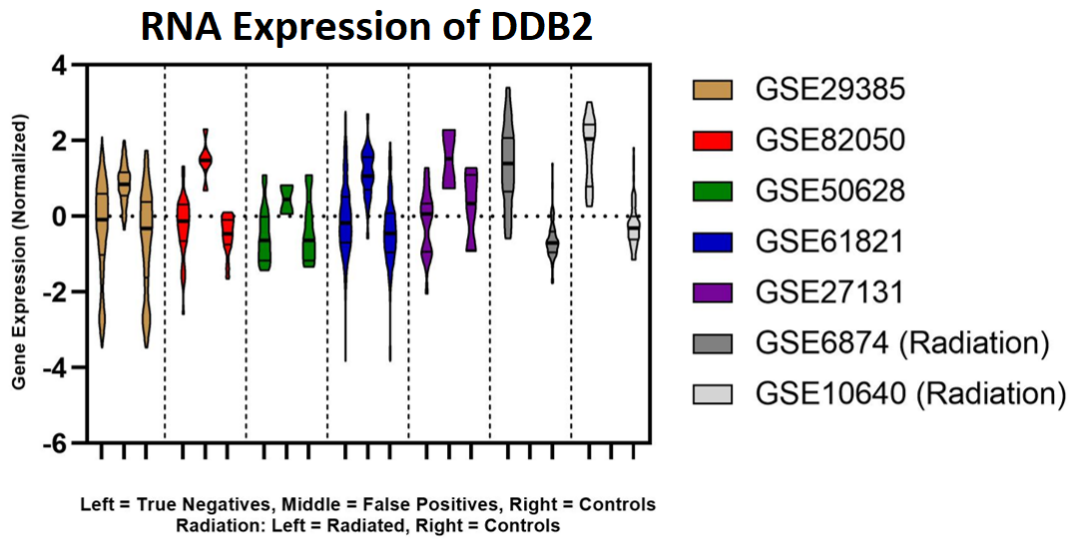
Violin Plots of *DDB2* Expression in Influenza- and Radiation-Exposed Patients. *DDB2* expression for Model 1 including Influenza patients, controls and radiation patients plotted using GraphPad. Each colour represents a different dataset. The left distribution of the radiation data (shaded grey) represents the expression of the radiated patients and the right distribution represents unirradiated controls. For all Influenza datasets (coloured), the left-most distribution represents the true negatives, the middle distribution represents the false positives, and the right-most distribution represents uninfected controls.

The four radiation gene signatures assessed from Zhao *et al.* ^48^ consist of 32 unique genes. When performing feature removal analysis (where model accuracy is reassessed after each gene is individually removed from it), 10 genes were identified that greatly contribute to patient misclassification: *DDB2*, *PCNA*, *GTF3A*, *PRKCH*, *CDKN1A*, *GADD45A*, *BCL2*, *MOAP1*, *TRIM22* and *TALDO1* (extended data ^39^ Section 1 – Table 8). *DDB2* is a DNA damage binding protein that is present in all four ML models. *DDB2* expression levels were elevated in irradiated patients, which is likely due a cellular response to radiation exposure, as this gene participates in nucleotide excision repair (it ubiquitinates histones H3 and H4 to increase accessibility of nucleosomes, exposing DNA and enabling access to XPC [xeroderma pigmentosum group C-complementing protein], which performs NER ^50, 51^). *DDB2* shared a similar pattern of expression between irradiated samples as well as infected patients that were misdiagnosed as irradiated (elevated *DDB2* expression in misclassified Influenza and Dengue patients; Figure 5). The activation of *DDB2* would be consistent with the proposed mechanism, whereby high levels of RNA viral genome increase the formation of abnormal, unresolved R-loops which in turn activate a DNA damage response. Expression of *DDB2* between those correctly classified and those misclassified as irradiated was deemed significant by the Mann Whitney test (p-value = 0.0001). Other genes with significant differences in expression included *GTF3A*, *PRKCH* and *PCNA* (which also has a role in the DNA damage response; extended data ^39^ Section 1 – Table 8).

### Biochemical kinetics of depleted RNA binding proteins in the human transcriptome

In the mechanism proposed ( Figure 1), the fraction of SRSF1 and RNPS1 bound to host RNA decreases as the fraction of SARS-CoV-2 genome increases as it replicates in the cell, causing RNA:DNA hybrids which result in R-loops. We therefore estimate the quantity of viral genomes and extent of viral replication required for viral binding site counts to approach, match, and exceed the number of host RNA sites available. These are derived from the number of SRSF1 and RNPS1 sites expressed in either a single A549 cell or a type II primary pneumocyte (cells were not infected; note that infection would be expected to alter the expression profile, which could affect expressed binding site estimates). The overall expression of each host gene was normalized by dividing by total expression of the given dataset, then by multiplying the number of all binding sites within a gene to its normalized gene expression value, and finally by multiplying the sum of all expression-adjusted binding site counts by the expected number of mature RNAs in a cell. We estimate a total of 80,000 RNAs per single cell (as determined by Marinov *et al.* ^52^), which is comparable with totals determined in other studies (e.g. Xia *et al.* ^53^ determined that a single osteosarcoma cell contains 92,000 ± 32,000 mature RNAs).

**Figure 6.**
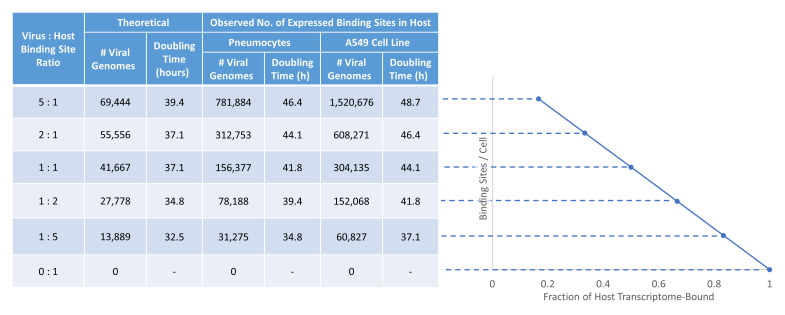
Inhibition of Host SRSF1 Binding by Viral Genome Replication. As the fraction of SARS-CoV-2 genomes increase in the host cell, the fraction of SRSF1 bound to the host transcriptome versus the viral genome decreases, resulting in R-loops. Strong SRSF1 binding sites (“Replicate 2” model) were identified in both SARS-CoV-2 (N=60 on the positive strand) and in the human transcriptome. A Scatchard plot (right) was created and used to determine the theoretical number of viral SRSF1 binding sites expected at different viral genome (inhibitor) to host (substrate) ratios (left).

Based on this approach, the total number of expressed binding sites (of any strength) was computed for SRSF1 and RNPS1 ( Table 1). However, this estimate includes sites expected to be weakly binding. When taking only strong binding sites into account, we estimate 12.7 to 18.2 million expressed SRSF1 (“Replicate 1” and “Replicate 2” SRSF1 models, respectively) and 9.9 million expressed RNPS1 binding sites in a single A549 cell. In a single primary pneumocyte, we estimated 6.6 to 9.4 million expressed SRSF1 sites (“Replicate 1” and “Replicate 2” models, respectively), as well as 5.2 million expressed RNPS1 binding sites. These estimates are based on expression levels in normal cells and may differ in infected cells. While the dissociation constant for RNPS1 is unknown, the dissociation constant of SRSF1 (K _d_) bound to the RNA sequence 5′-UCAGAGGA-3′ has been experimentally measured as 0.2 μM ^54^. With the K _d_, a Scatchard plot for SRSF1 binding was derived where host bind sites are substrates and viral binding sites are considered to be inhibitors of host RNA binding. We assumed no free RNA binding protein (that the vast majority of SRSF1 is bound to either host or viral binding sites) as the concentration of free RBPs is likely to be low due to sequestration of RBPs by the excess of viral sequences present in infected cells (~60% of all RNA ^55^). This assumption is reasonable for strong binding sites (where *R _i_* ≥ *R _sequence_*). We use K _d_ to compute the theoretical number of viral genomes required to satisfy various viral genome to host binding site ratios ( Figure 6 [Table left]). This calculation is also carried out without reference to K _d_, by instead computing the number of viral genomes required to achieve binding site ratios in viral to host-bound RBP from a direct analysis of primary pneumocyte and A549 transcriptomes. The number of strong SRSF1 binding sites in a single viral genome multiplied by the level of viral replication is compared with the estimated number of expressed SRSF1 sites in the host nucleus (in a pneumocyte or an A549 cell; Figure 6 [Table right]). The data presented in Figure 6 uses the number of sites predicted by SRSF1 “Replicate 2” model, and only considers the positive strand of SARS-CoV-2. Despite their similarities, the SRSF1 “Replicate 2” model predicts far more binding sites on the positive strand of SARS-CoV-2 compared to the “Replicate 1” model (N=60 and 31, respectively). This leads to small differences in the estimated doubling time, when only the positive strand of the virus (extended data ^39^ Section 1 – Table 9A) is considered. An examination of potential binding sites on both strands of SARS-CoV-2 does not appreciably alter the estimated doubling time for both SRSF1 IWMs (extended data ^39^ Section 1 – Table 9B).

The doubling times required for infection initiated by a single virion were computed for varying numbers of viral genomes, as replication increases the overall counts of viral RBP binding sites. The processivity rate of genome replication for SARS-CoV-2 is currently unknown, so a value was estimated based on a polymerization rate of 3.7 nt/s for a different RNA-dependent viral RNA polymerase, that of Vesicular Stomatitis Virus (VSV) ^56^. The doubling time was then adjusted to 2.31 hours per replication event, based on the increased length of the SARS-CoV-2 genome (L=30,899nt) compared to VSV. The doubling time is estimated to be between 37.1 to 44.1 hours to achieve a level of SARS-CoV-2 binding that depletes RBP from an equal number of expressed host nuclear RNA sites (1:1 ratio). However, fewer replication events and shorter doubling times are computed using the published K _d_ of SRSF1 (between 5-9 hours less). The number of replication events required for viral genome binding sites to overtake host RNA binding was less in primary pneumocytes compared to A549 cells (~2.3 hours or 1 doubling of the SARS-CoV-2 genome). This was anticipated, since the total number of expressed SRSF1 (and RNPS1) sites are lower in primary pneumocytes than the immortalized cell line due to lower overall gene expression levels.

## Discussion

We propose a previously undescribed putative mechanism of RNA viral infection-induced apoptosis, supported RNA binding events determined by information theoretic analysis. In the mechanism, viral release is enhanced by viral genome replication, which sequesters RBPs, thereby depleting native binding of RBPs to and stabilization of host-encoded transcripts. This process can occur in either the cytoplasm or the nucleus of the host cell, depending on specific replication requirements of different viral families. In SARS-CoV-2, this is expected to substantially reduce import of RBPs into the nucleus. Reduced availability of nuclear RBPs promotes R-loops through formation of complimentary duplexes between nascent transcripts and chromosomal sequences. High densities of R-loops at a late stage of infection would be expected to overwhelm cellular DNA repair mechanisms that ordinarily remove these structures and eliminate DNA breakage. DNA damage markers *DDB2* and *PCNA* are increased in both Influenza and Dengue infections, respectively. Unrepaired, persistent chromosome double strand breaks are unstable and induce apoptosis, which would be expected to release high viral titers.

We utilized a well-established information theory-based approach to demonstrate the validity of this proposed mechanism ^17– 24^. IT-based models of RBP binding sites was used to scan viral RNA genomes (Influenza, SARS-CoV-2 and Dengue) and host transcriptomes. IT models derived from thousands of validated RBP binding sites delineated numerous strong SRSF1, RNPS1 (and hnRNP A1) RNA binding sites within these viral genomes. The derived SRSF1 and RNPS1 binding motifs were shown to be highly similar, consistent with previous published studies demonstrating that RNPS1 could partially complement genomic instability due to SRSF1 deficiency. Indeed, both models detected many of the same RNA binding sites in the host transcriptome and all strong RNPS1 binding sites detected in the SARS-CoV-2 genome were simultaneously detected by at least one SRSF1 information model. In divergent strains of both SARS-CoV-2 and Influenza A (H3N2), the frequencies and strengths of these binding sites are highly consistent. Finally, we estimate that the quantity of replicated viral genomes necessary to meet or exceed the number of binding sites expressed within a lung can exceed the site counts in the host genome, and the doubling time required to deplete these RBPs which is consistent with the observed time course of severe infections.

The estimated doubling times were based on the assumption that the RNA polymerization rate of SARS-CoV-2 was similar of that of VSV. However, the replication rate of RNA dependent RNA polymerase of the original SARS-CoV virus is considerably faster (600-700 nt/s ^57^). The similarity between these coronaviral sequences implies that the SARS-CoV-2 genome might replicate in under a 1 minute. If the viral replication rate in our study from VSV is an underestimate, the corrected processivity of this enzyme would be expected to accelerate sequestration of RBPs, as well as the proposed R-loop formation and apoptosis. However, the polymerization rate measured *in vitro* may not be sustainable due to reaction *in vivo* constraints (e.g. nucleotide pool depletion and subcellular compartmentalization).

Functional analyses will be needed to prove that this mechanism plays a role in viral pathogenicity. Such studies should further investigate how infections of SARS-CoV-2 (and other RNA viruses) cause increased DNA damage. RNAseq and protein expression analysis of *DDB2*, *RAD17*, *PRKDC*, *PCNA* and other ATR pathway markers of infected cells accompanied by time course studies of nascent double stranded chromosomal breaks (i.e. H2AX antibody staining due to viral infection) would provide such evidence. Increased R-loop formation upon infection will be required, with particular attention to host encoded transcripts enriched in SRSF1 and RNPS1 binding site clusters. Although the genomic coordinates where R-loops form can be anticipated from information dense clustering, the strand- and gene specific techniques used to detect these, i.e. DRIPc-Seq, cannot measure RNA-DNA hybrids of lengths shorter than 70bp ^46^. Sequence-based chromatin immunoprecipitation with antibodies to H2AX, 53BP1 or other markers of DNA damage should be consistent with the sites of R-loop formation. Changes in the expression of apoptotic markers (e.g. *BCL2*, *BCL2L2*, *BAX*, and *TNFRSF10B*) would also be expected in infected cells with high levels of replication. Direct interaction between RBPs and viral genomes must also be demonstrated, possibly by immunoprecipitation or copackaging in viral capsids. It should also be possible to evaluate the possibility that inhibitors of viral replication, such as remdesivir (and any other nucleoside analogs), can reduce DNA damage, R-loop formation, and apoptosis of infected cells.

SARS-CoV-2 efficiently infects multiple species of mammals ^58^, and possesses an RNA polymerase with proofreading capability, which enables it to faithfully and accurately replicate and transcribe its genome. In this study, we suggest that effects of SARS-CoV-2 infection are mild in most individuals because most of us mount robust immune responses and eventually clear the virus. The mechanism that we propose ( Figure 1), which may be a contributing factor of a variety of different RNA viruses, has the potential to overwhelm that response through jackpot replication coupled to apoptotic events caused by loss of chromosome integrity stemming from depletion of essential RBPs. This results in high multiplicities of infection of cells in the most vulnerable cells. This could cause a rapid onset of loss of viable pneumocytes, and compromising oxygen transport, to a point where it is insufficient to maintain blood pO _2_ levels to support organ functions. Systemic inhibition of viral replication and transcription of viral proteins will be essential to prevent or mitigate this pathological mechanism.

Other coronaviruses such as MERS and SARS have been shown to induce apoptosis ^59^. The polyphenol Resveratrol has been shown to downregulate apoptosis *in vitro* ^59, 60^, possibly by overexpressing sirtuins (a family of signalling proteins). However, this is ultimately not a practical solution to infection, as the drug will only delay an eventual high multiplicity infection event. In order to inhibit the viral mechanism proposed in this study, a drug must inhibit the viral machinery that sequesters spliceosomal components, leading to R-loops and DNA damage. This may explain, in part, why remdesivir (Gilead) improves the recovery of SARS-CoV-2 patients. The drug, which was originally developed for treatment of Ebola virus by inhibiting its RNA dependent RNA polymerase, also inhibits viral replication of SARS-CoV-2. Other potential therapies include those targeting expression of genes encoded by the viral genome, which use a common 5’ leader sequence of all transcripts. The promoter sequence for these genes binds to the host encoded hnRNP A1, which regulates transcription of beta coronaviral genes (of which SARS is a member of that family). While hnRNP A1 could be a potential drug target for therapy (there are small molecules that have been shown to inhibit hnRNP A1 RNA splicing activity ^61^), there would be concerns that this may cause inadvertent side effects due to its impact on normal mRNA splicing.

Regardless of whether apoptosis releases large quantities of mature infectious virus, the proposed mechanism will still likely impact pneumocyte function. Should high multiplicities of infection be the result of apoptotic release of virions, then the proposed RBP depletion mechanism would be expected to kill both the original infected cell and neighboring infected pneumocytes. The severe symptoms might be the result of rapid, overwhelming lysis of cells responsible for oxygen transport, rather than by a cytokine storm. Autopsies of infected individuals from Wuhan China have shown evidence of inflammation, but not necessarily macrophage invasion and pulmonary edema ^62^. Furthermore, apoptosis has been demonstrated in lung epithelial cells in Macaques infected with Influenza virus ^63^. This could explain why physicians and other health professionals in repeated contact with multiple infected patients do not seem to have time to develop immunity to the virus, regardless of their age. Type II pneumocytes which produce surfactant, required at high levels in newborns, decrease with age ^64^ and are particularly diminished in individuals with respiratory disease like COPD (Chronic obstructive pulmonary disease) and ARDS (Acute respiratory distress syndrome). If the multiplicity of infection (MOI) of virus damages this population of cells, then individuals with fewer cells might be more susceptible to exhibiting insufficient pulmonary function due to the high MOI released by the mechanism proposed. These patients would be at greater risk for severe complications requiring assisted ventilation. It is also possible that the deficiency of functional pneumocytes in such individuals cannot be compensated for by extracorporeal membrane oxygenation to rescue multiple organ failure.

Humans have high numbers of type II pneumocyte cells at birth to fulfill demands for surfactant to rapidly expand lung volume. Synthetic surfactant is an essential treatment for premature birth, since these cells mature late in gestation. Age-related loss of these cells has been measured and the mechanism leading to it was described ^64^. Loss of functional pneumocytes is particularly evident in individuals with ARDS, who exhibit significant lung fibrosis, which is also seen in patients with SARS-CoV-2 infections. Older individuals (or those with pre-existing respiratory conditions) are more susceptible to the loss of the remaining cells by apoptosis or autophagia. Decreased pneumocyte counts affect O _2 _transport efficiency, which lowers blood pO _2_, and extant tissues and organs. The proposed mechanism implies that jackpot viral replication events, regardless of age of the infected individual, enhances viral release through apoptosis and infection. Such events are more likely in cells infected by coronaviruses like SARS-CoV-2, which are capable of repressing the innate immune response, i.e. induction of interferon response to viral double stranded RNA (unlike Influenza) ^55, 65, 66^. Repression of innate immunity enables the virus to replicate unabated in these cells, which would be expected to delay their recognition by regulatory T cells and killing by macrophages.

Viral infections significantly alter the transcriptional profiles of host genes in infected cells. Recent studies of Zika virus (an RNA virus) have revealed that infection not only impacts transcription, but affects alternative mRNA splicing as well ^67^. Both RNA and DNA viral infections encode factors that directly ^68^ or indirectly ^67^ alter host RNA processing, resembling alternative mRNA isoforms. We suggest that the mRNA splicing changes observed subsequent to infection of an RNA virus could be a consequence of replicated viral genome binding to RBPs, thus changing the nuclear stoichiometry of splicing proteins (such as SRSF1). This would effectively reduce the concentration of available splicing factors, which could be responsible for the observed alternative splicing events of other splicing factors (such as SRSF2 and SRSF3) reported by Bonenfant *et al.* ^67^. Thus, the mechanism proposed in this study may not only impact genome stability by the introduction of R-loops, but may simultaneously alter the global alternative splicing landscape in infected host cells.

RNA-based vaccines based upon synthetic SARS-CoV-2 transcripts containing modified nucleosides that have been dephosphorylated to escape innate immunity are being tested ^69^. These candidates exploit host protein synthesis machinery to transiently express viral antigens that activate B and T-cell immunity. However, these synthetic RNAs would also be available for RBP binding. A transcript encoding the SARS-CoV-2 spike glycoprotein ‘S’ gene, for example, would contain 7 strong RNPS1 and between 6 to 8 strong SRSF1 binding sites (depending on SRSF1 model). If the levels of expression produced from these transcription templates cannot be carefully controlled, excess production of these RNAs could potentially elicit undesirable side effects through sequestration of critical host RNA binding proteins required to inhibit R-loop formation.

Localization of viral replication to the cytoplasm does not obviate the fact that there is still a competition between the host and viral genomes for these RNA binding proteins. While the binding site stoichiometry calculations are unchanged, compartmentalization of the viral and host genomes does have implications for preventing R-loops during host transcription. Since coronavirus replicates in the cytoplasm, binding of newly synthesized RBPs occurs there. This makes less protein available to be imported into the nucleus for binding to nascent transcripts to prevent R-loops from forming. The viral genome may have an advantage in this competition for binding to RBPs relative to nuclear transcripts, due to the proximity of the viral genome to nascent RBPs in the cytoplasm, which may limit transport and impede their import into the nucleus. RBPs are often highly expressed, including SRSF1 and RNPS1, and are abundant in the lung (where SARS-CoV-2 infection is most prominent). Thus, the cytoplasmic concentration of viral genome necessary to prevent the localization of RBPs into the nucleus is likely to vary between different tissues.

The proposed mechanism of RNA virally-induced apoptosis is supported by extensive bioinformatic analyses indicating that strong RNA binding sites of host RBPs are common in RNA viral genomes, and that the frequencies of such binding sites are relatively consistent between divergent strains in both Influenza A and SARS-CoV-2. Future efforts should elucidate details of the mechanism with functional analysis of infected cells, including demonstration of increased R-loop formation, induction of relevant apoptotic or DNA repair responses, and direct interaction between viral genomes and host RBPs. This would justify further investigations into binding of specific RBPs to viral sequences in infected patients. The potentially prognostic significance of such data could be useful in differentiating among drug therapies that target RNA viral genome replication and/or expression.

## Methods

### Information theory-based RNA binding site analysis

The IWMs for the RBPs investigated in this study (SRSF1, RNPS1 and hnRNP A1) were either obtained for previously published analyses or derived in this study. The hnRNP A1 IWM used in this study was previously derived in Peterlongo *et al.* (using PoWeMaGen software [v1] ^22^) using an hnRNP A1 CLIP-seq dataset ^70^. The functionality provided by PoWeMaGen is also available in Delila software, which is open source. IWMs can also be derived with the ‘ *Ri’* program, and RBP binding sites can be localized with the ‘ *Scan*’ program of the Delila package. Individual binding site strengths ( *R _i_* values) of these IWMs can also be determined using the ‘ *Scan*’ program.

A previously described IWM for SRSF1 ^21^ was based on only 28 manually curated and validated and aligned binding sites ^71^. To update this IWM, we derived new SRSF1 models from high-throughput eCLIP datasets containing thousands of validated binding sites of 150 different RBPs ^34^. Narrow peak files from two separate SRSF1 eCLIP replicates ( ENCFF179SCM and ENCFF184TBM), as well as two non-target, negative control replicates ( ENCFF241ORF and ENCFF773PUP) were retrieved from the ENCODE Data Coordination Center (ID: ENCSR456FVU) ^34^. The new SRSF1 and negative control SRSF1 IWMs were generated using Maskminent v1.0.2 ( 24; https://doi.org/10.5281/zenodo.49234). Both PoWeMaGen and Maskminent utilize the Bipad algorithm to align binding sites ^72^. Similarly, RNPS1 and GFP-control IWMs were derived from publicly available iCLIP data ( 31; E-MTAB-4215). However, this iCLIP dataset was only available in FASTQ file format, which required further processing to identify CLIPseq peaks. Thus, the available RNPS1 iCLIP data was first aligned to the human genome (GRCh37) with TopHat v2.1.1, and then converted to peaks using Piranha v.1.2.1 (a CLIP- and RIP-seq peak caller) under default settings.

IWMs for SRSF1 and RNPS1 were derived from eCLIP and iCLIP-seq datasets (respectively) using Maskminent under varying model length conditions (6-10nt long; 1,000 Monte Carlo cycles). As experimental noise has been found to contribute to non-specific IWMs ^24^, we limited model derivation to only the to the 5,000 or 50,000 iCLIP peaks with the highest signal value (SRSF1) or the lowest p-values (RNPS1; computed by Piranha). In practice, the derived models remained similar regardless of the size of peak subset used. As many intervals from the SRSF1 and RNPS1 datasets were short (<20nt), peak lengths were extended on either direction by the sequence length (e.g. a 10nt interval becomes 30nt long). We found that both RNPS1 and SRSF1 models derived at lengths of 6nt to be most informative with similar *R _i_* densities, although they differed slightly ( Table 1). Both the RNPS1 model and the SRSF1 model derived from the second replicate (SRSF1 “Replicate 2”) selected was generated from 5,000 CLIP-seq peaks, while the SRSF1 “Replicate 1” model was derived from 50,000 peaks.

To evaluate the similarity between these IWMs, the RNPS1 and SRSF1 motifs were compared using the STAMP web server ^35^, which performs a pairwise alignment between each motif (ungapped Smith-Waterman alignment method) and compared using a Pearson correlation coefficient distance metric, and outputs results as e-values. Statistical significant differences between the e-values of IWMs for RBPs were compared with their corresponding negative control motifs. These were quantified as log10 likelihood ratios determined from the pairwise RBP motif comparison relative to the same RBP with its negative control motif e-values according to:

$$\text{LOD score}\;\text{=}\;{\text{log}}_{\text{10}}\left(\frac{e-value\;\left(RBP\;vs.\;RBP\;IWM\right)}{e-value\;\left(RBP\;vs.\;neg.control\;IWM\right)}\right)$$

### Transcriptome, exome and viral genome RBP scans

The human reference genome (GRCh37; Genbank Acc. GCA_000001405.1; downloaded from UCSC [ https://hgdownload.soe.ucsc.edu/goldenPath/hg19/bigZips/]) and viral genomes (Dengue virus 3 [GenBank accession: NC_001475.2]; human immunodeficiency virus type 1 [HIV-1] HXB2 [Genbank: K03455.1] and subtype C [Genbank: U46016.1]; Influenza A H3N2 strains [Ontario/104-25/2012; Genbank (segments 1-8): KJ413878.1, KJ413896.1, KF840477.1, KJ413897.1, KJ413880.1, KJ413864.1, KJ413915.1, KJ413925.1] and [Shanghai/C84/2009 Genbank (segments 1-8): JX286598.1, JX286597.1, JX286596.1, JX308801.1, JX286594.1, JX286593.1, JX286592.1, JX286595.1]; and SARS-CoV-2 [Genbank: NC_045512.2]) were scanned with each IWM (SRSF1 [two separate models], RNPS1 and hnRNP A1). Human genome scans were then filtered so that only those predicted binding sites found in transcribed regions (using the Ensemble Genes database [release 99]) would be considered. Only sites exceeding *R _sequence_*, the average information content of the binding site model, were retained in subsequent analyses as these consist of mean binding affinity or higher and are likely to more effectively compete for binding to these proteins ^24, 25^. The *R _sequence_* values of each model are: 6.7 bits (SRSF1 “Replicate 1” model), 6.4 bits (SRSF1 “Replicate 2” model), 7.8 bits (RNPS1 model), and 4.6 bits (hnRNP A1 model).

Besides those previously indicated, viral genomes of multiple other SARS-CoV-2 and Influenza A (H3N2) strains were scanned using the IWMs for SRSF1, RNPS1 and hnRNP A1 to evaluate whether divergent strains of these viruses carry significantly different strong binding sites counts. NextStrain (which provides real-time tracking of the SARS-CoV-2 and Influenza A) was utilized to choose divergent strains of either virus by selecting strains from separate clades, i.e. different monophyletic groups. The viral genome sequences of selected SARS-CoV-2 strains (Genbank accessions: MT007544.1 [Australia], MT066176.1 [Taiwan], MT121215.1 [China], MT163718.1 [USA], MT188339.1 [USA],MT198652.1 [Spain], and MT198653.1 [Spain]) and Influenza A H3N2 (GISAID accessions: EPI1676017-EPI1676024 [Denmark], EPI1635542-EPI1635549 [England], EPI1594883-EPI1594890 [Singapore], EPI1614613-EPI1614620 [Sydney]) were downloaded from the GISAID database. Each of these genome sequences were evaluated for strong SRSF1, RNPS1 and hnRNP A1 binding sites. All binding sites (with *R _i_* ≥ *R _sequence_*) are provided in extended data ^39^, Section 1 – Tables 3 and 4.

### Expressed RNA binding sites in lung cells

Publicly-available expression datasets were downloaded from the Gene Expression Omnibus for A549 cell lines ( GSE141171; RNAseq) and primary type II pneumocytes ( GSE86618; scRNAseq). Normal expression for each cell type was computed by taking the average of all control samples from each dataset (N=3 control samples in GSE141171; N=215 control samples in GSE86618). We then use this information to estimate the total number of binding sites present in a single pneumocyte or A549 cell. First, the program “ScanDataSummaryProgram.pl” (available within underlying data ^39^ Section 6) was used to compute the total number of binding sites (≥ *R _sequence_*) in each cell type for each expressed gene (TPM >0; underlying data ^39^ Section 1 - Table 5). The overall expression of each gene was then normalized using the program “TotalBindingSitePerCellCalculator.pl” (underlying data ^39^ Section 6), which divides expression by the sum of all TPM values in the cell, multiplied by the estimated number of mature RNAs in a cell at any given timepoint (80,000 RNAs per lymphoblastoid cell ^52^). It then multiplies this normalized gene expression value with its binding site total to determine the overall contribution of binding sites from that gene in a single cell. The sum of this value across all expressed genes gives the total number of RNA binding sites expected to be available in a cell at any given time ( Figure 6).

### Information-dense clustering of RBPs across viral genomes and human transcriptome

Information dense clustering has previously been applied to the human genome to identify clusters of organized TFBSs ^25, 40^. The clustering software (v1; described in reference 25; software provided in a Zenodo archive - https://doi.org/10.5281/zenodo.1707423) was used in this study to identify clusters of low-affinity ( *R _i_* > 0 bits), moderate-affinity (≥ $\frac{1}{2}$ *R _sequence_*) and high-affinity (≥ *R _sequence_*) RBP sites in both the viral genomes investigated in this study, and across the entire human transcriptome. To be considered a cluster, each set of component sites was required to occur ≤25nt from one other, and the total information of all sites within the cluster equalled or exceeded ≥50 bits. In its original design, the clustering algorithm considered binding sites on both strands in forming clusters. To maintain strand specificity, we separated input by strand. Due to the high memory demands of the clustering algorithm, transcriptome scan input was separated into segments of ~200,000 sites per run, which was then subsequently combined. To avoid the inadvertent separation of a binding site cluster, input was split only when two sequential binding sites were >1,000nt apart.

### Identification of RBP sites and clusters within DRIP-seq intervals

All binding sites and information-dense clusters identified in the human genome were intersected with DRIP-seq and DRIPc-seq intervals, which indicate where there is evidence of R-loop formation in the human genome (performed by “ClusterToDRIPseqAnalysisProgram.pl”; underlying data ^39^ Section 6). The DRIP-seq dataset ( GSE68845; IMR90 cells) is not strand specific, thus binding sites and clusters from either strand are considered when intersected against these intervals. DRIPc-seq data ( GSE70189; NTERA2 cells), however, is strand specific which has been taken into account (e.g. positive strand clusters found in positive strand DRIPc-seq intervals reported). We then computed the gene density of sites and clusters that are found within these intervals (underlying data ^39^ Section 1 - Table 5) using the script “ClusterToDRIPseqAnalysisProgram.GeneDensityFinder.pl” (underlying data ^39^ Section 6) to determine if there is a correlation between the presence of binding sites and R-loop formation.

### Lollipop plots and intersite distance histograms

Lollipop plots which indicate the location of information-dense clusters for all viral genomes described in this study and for all genes in the human transcriptome (with ≥1 cluster) were generated in R (version 3.6.3) using the Bioconductor package “ trackViewer” (v.1.20.3 ^73^). The lollipop plots presenting human genes contain intron and exon boundary information which was generated using the RefSeq database (release 60). Multiple lollipop plots were generated for multi-segmented viral genomes (one image per segment). The height of each “lollipop” corresponds to the information density of a cluster, and its location in the genome is indicated (GRCh37) along with the number of sites which comprise the cluster.

Histograms which illustrate the distribution of binding site *R _i_* values and the frequency of the distance between RBPs (“intersite distances”) were generated using the R package ‘ ggplot2’ (v3.1.1 ^72^). Intersite distance frequency was determined by first grouping all RBP by gene, followed by determining the distance between each site in sequential order. Distance thresholds of 500nt or 1,000nt were assigned for all intersite distance histograms. Rare instances of distances greater than these thresholds were excluded from the histogram, as their inclusion led to plots too wide to be informative.

### Radiation gene expression signatures and viral infection

Gene expressions for individuals with the diseases above were collected from Gene Expression Omnibus (GEO), which consisted of 5 Influenza studies ( GSE29385, GSE82050, GSE50628, GSE61821, GSE27131), 4 Dengue studies ( GSE97861, GSE97862, GSE51808, GSE58278) and 2 studies involving Aplastic Anemia patients ( GSE16334, GSE33812). We also collected expression data from two studies with radiation-exposed samples ( GSE6874 and GSE10640). The best performing human signatures (assessed by traditional validation; described in Table 7 [underlying data ^39^ Section 1]) from Zhao *et al.* ^48^ were then used to test the gene expression datasets in order to determine if these models would misclassify infected patients as irradiated (with and without control patients). Models were tested using the MatLab script used to perform “traditional validation” in the Zhao *et al.* study (“regularValidation_multiclassSVM.m”, https://zenodo.org/record/1170572), which first normalizes gene expression values by quantile normalization before applying the radiation model to the infected patient data to predict outcome. The script then compares prediction of radiation exposure to the clinical data provided. MatLab scripts are compatible with GNU Octave.

To better understand why the radiation models are predicting certain Influenza- and Dengue-infected patients as irradiated, violin plots were generated using GraphPad Prism v8 to visually illustrate differences in gene expression between infected individuals correctly classified and those misclassified by each radiation model ( Figure 5). When inspecting violin plots of the 32 genes which make up the 4 radiation models tested, 10 genes were identified to have contributed towards false positives predictions as they shared a similar pattern of expression in those that were radiated in two gene expression datasets of irradiated individuals (GSE6874 and GSE10640). The 10 genes are: *DDB2*, *PCNA*, *GTF3A*, *PRKCH*, *CDKN1A*, *GADD45A*, *BCL2*, *MOAP1*, *TRIM22* and *TALDO1*. Mann-Whitney tests were used to compare the expression of these genes in false negative and true positive patients. Four genes ( *DDB2*, *PCNA*, *GTF3A* and *PRKCH*) were consistently found significant in most of the studies tested.

### Association kinetic analysis

The dissociation constant of SRSF1 bound to the RNA sequence 5′-UCAGAGGA-3′ was experimentally determined to be 0.2 μM ^54^. This information allowed for the derivation of a theoretical Scatchard plot for SRSF1 binding by varying the relative proportions of viral to host binding sites bound (where viral binding sites are considered inhibitors, and host binding sites as substrate). We can compute the theoretical number of viral genomes necessary to reach these relative proportions according to:

$$\frac{v}{\left[L\right]}=\frac{n}{K_{d}}-\frac{v}{K_{d}}$$

Where K _d_ is the SRSF1 dissociation constant, n is the number of sites (or sequences) that a single protein can bind (n=1), [L] is the concentration of free SRSF1, and v is the amount of SRSF1 bound to the viral genome relative to host. Upon infection and viral replication, it is assumed there is no free RNA binding protein (all RBP is assumed to be bound to either viral or host RNA). These proportions were converted to numbers of viral genomes per infected host cell (determined using the above formula in an MS- Excel spreadsheet), adjusted for the computed number of viral genomes per cell by the number of SRSF1 binding sites in a single viral genome (described earlier). We also computed the number of viral genomes necessary to reach these proportions by taking A549 or pneumocyte host cell binding site expression (computed previously) into account. We then used the published processivity rate of 3.7 nucleotides/sec for VSV RNA dependent RNA polymerase ^56^ to estimate the doubling time required.

## Statistical analysis

The average distances between adjacent binding sites of SRSF1, RNPS1 and hnRNP A1 were determined within both expressed human genes and RNA viral genomes (Dengue, HIV-1 strains B and C, Influenza A and SARS-CoV-2). A program script “calculateIntersiteDistance.pl” (underlying data ^39^ Section 6) takes a set of binding site coordinates and their associated genes as input and determines the pairwise distances between all consecutive binding sites in the same gene. Subsequently, “removeOutliersHigherThanN.pl” is used to discard extreme outlier distances exceeding a specified threshold (thresholds of 500nt and 1,000nt were evaluated). Finally, “getStatisticsOnCol.pl” evaluates a given set of intersite distances and computes the count, geometric mean, median, arithmetic mean and their standard deviation. The program was used to evaluate intersite distances at multiple *R _i_* thresholds (low- [ *R _i_* > 0 bits], moderate- [≥ $\frac{1}{2}$ *R _sequence_*] and high-affinity [≥ *R _sequence_*] binding sites). We also examined binding sites which intersect DRIPc-seq intervals in the human genome using this procedure. Output from this analysis are provided as histograms in extended data ^39^ Section 5, as described earlier.

## Data availability

A data repository titled “Characteristics of human and viral RNA binding sites and site clusters recognized by SRSF1 and RNPS1” has been deposited as a Zenodo archive (DOI: 10.5281/zenodo.3737089 ^39^). The archive contains the following underlying and extended data, organized across 6 sections. Section 1 primarily consists of extended data, and Sections 2–6 contains the underlying data presented in the paper.

### Extended data

Zenodo: Characteristics of human and viral RNA binding sites and site clusters recognized by SRSF1 and RNPS1. http://doi.org/10.5281/zenodo.3737089 ^39^

This project contains the following extended data:

Section 1 – The nine additional tables described in this study (“Section 1 - Tables 1–9”), which provide SRSF1, RNPS1 and hnRNP A1 binding site and information-dense cluster counts across various RNA viral genomes [including multiple SARS-CoV-2 and Influenza strains] and the human transcriptome, the estimated SARS-CoV-2 doubling time necessary for viral genome SRSF1 binding site availability to exceed sites within the host transcriptome, and an analysis of Influenza, Dengue, and aplastic anemia patients misdiagnosed as irradiated by established radiation gene signatures.

### Underlying data

Zenodo: Characteristics of human and viral RNA binding sites and site clusters recognized by SRSF1 and RNPS1. http://doi.org/10.5281/zenodo.3737089 ^39^

Section 2. All SRSF1, RNPS1 and hnRNP A1 binding site genome browser tracks for human and all viral genomes analyzed in this study (GRCh37).

Section 3. The full set of lollipop plots (indicating the location of SRSF1, RNPS1 and hnRNP A1 information-dense clusters) in all human genes and in each of the viral genomes analyzed.

Section 4. The Ri(b,l) matrices or IWMs for all RBPs analyzed (SRSF1, hnRNP A1 and RNPS1).

Section 5. The full set of histograms which display the distribution of *R _i_* strength and intersite distance between the binding sites for each RBP [across all transcribed regions or within known DRIPc-seq intervals.

Section 6. A set of 7 Perl scripts created specifically for this study, with instructions for their use: A) “ClusterToDRIPseqAnalysisProgram.pl” – reports which information-dense clusters are located within DRIPc- and/or DRIP-seq intervals (individually and by gene); B) “ClusterToDRIPseqAnalysisProgram.GeneDensityFinder.pl” – uses the output from script “A” to determine the number and the density of information-dense clusters within a gene (total clusters within the gene and those within DRIPc-seq intervals); C) “calculateIntersiteDistance.pl” – determines the distance between all binding sites in the same gene from a list of genomic coordinates; D) “removeOutliersHigherThanN.pl” – discards intersite distances computed by script “C” that are greater than a specified threshold; E) “getStatisticsOnCol.pl” – calculates the count, geometric mean, median, arithmetic mean, and standard deviation of values from script “D”; F) “ScanDataSummaryProgram.pl” – determines the number of binding sites (above a specified *R _i_* threshold) found within known genes (the program also reports the total expression of those genes using external A549 and pneumocyte expression datasets) from binding site coordinate data; G) “TotalBindingSitePerCellCalculator.pl” – estimates the number of binding sites expressed in a single A549 or pneumocyte cell at any given time.

Data are available under the terms of the Creative Commons Attribution 4.0 International license (CC-BY 4.0).
