# Supplementary material for: A proposed molecular mechanism for pathogenesis of severe RNA-viral pulmonary infections
Source: F1000Res. 2021 Jan 6;9:943. Originally published 2020 Aug 7. [Version 2] doi: 10.12688/f1000research.25390.2 (PMC7676395; doi:10.12688/f1000research.25390.2)
Supplement: Peer review history

# Peer review: Reviewer response for version 2

**DOI:** 10.5256/f1000research.31422.r76718

**Published:** 2021-01-07

**Authors:** Gregory Fonseca

**Affiliations:** 1 Division of Experimental Medicine, Department of Medicine, McGill University, Montreal, Canada

The authors have answered my concerns.

Is the work clearly and accurately presented and does it cite the current literature?

Yes

If applicable, is the statistical analysis and its interpretation appropriate?

I cannot comment. A qualified statistician is required.

Are all the source data underlying the results available to ensure full reproducibility?

Yes

Is the study design appropriate and is the work technically sound?

Yes

Are the conclusions drawn adequately supported by the results?

Yes

Are sufficient details of methods and analysis provided to allow replication by others?

Yes

Reviewer Expertise:

Bioinformatics, transcriptomics, genomics. Lung disease.

I confirm that I have read this submission and believe that I have an appropriate level of expertise to confirm that it is of an acceptable scientific standard.

# Peer review: Reviewer response for version 2

**DOI:** 10.5256/f1000research.31422.r76719

**Published:** 2021-01-06

**Authors:** Ian Eperon

**Affiliations:** 1 Leicester Institute of Structural & Chemical Biology, Department of Molecular & Cell Biology, University of Leicester, Leicester, UK

No further comments.

Is the work clearly and accurately presented and does it cite the current literature?

Yes

If applicable, is the statistical analysis and its interpretation appropriate?

Yes

Are all the source data underlying the results available to ensure full reproducibility?

Yes

Is the study design appropriate and is the work technically sound?

Yes

Are the conclusions drawn adequately supported by the results?

Yes

Are sufficient details of methods and analysis provided to allow replication by others?

Yes

Reviewer Expertise:

RNA splicing.

I confirm that I have read this submission and believe that I have an appropriate level of expertise to confirm that it is of an acceptable scientific standard.

# Peer review: Reviewer response for version 1

**DOI:** 10.5256/f1000research.28014.r73886

**Published:** 2020-11-26

**Authors:** Ian Eperon

**Affiliations:** 1 Leicester Institute of Structural & Chemical Biology, Department of Molecular & Cell Biology, University of Leicester, Leicester, UK

The purpose of this research is to establish by computational methods whether the genomes of certain RNA viruses could provide enough binding sites for proteins that bind nascent transcripts to reduce their availability in the cell and thereby trigger R-loop formation and ultimately apoptosis. The authors focussed their attentions of SRSF1 and RNPS1, both of which are known to prevent R-loops.

 The first part of the work involved a derivation of a new information weight matrix from ENCODE eCLIP datasets. I am unable to comment on the methods used, but it is reassuring that the resultant consensus sequences for SRSF1 matched a coalescence of previous results from SELEX, RNA-seq and structural work. The authors then analysed the occurrence and distribution of all motifs in both viral and genomic transcripts that had a higher information content than the mean of the information content for all the binding sites in the model for each protein. It was assumed, but has not yet been tested, that the sites elected are the stronger binding sites. It is not clear whether this has any biological relevance, i.e., how bound lifetimes (affinity) vary across all the sequences in the model and whether the threshold chosen is likely to reflect the real behaviour of the proteins. Nonetheless, in the context of this heuristic work, this is not an unreasonable choice to make.

 The authors analysed in particular the occurrence of clusters of these sites, i.e., where sites were within 25 nts of each other, and they looked for a correspondence between the locations of these clusters and sites located by high-throughput methods at which it was known that R-loops are likely to form. This correspondence was not strong, although the binding sites for RNPS1, SRSF1 and hnRNP A1 were closer than average in these regions. This is followed by a comparison of gene expression in patients with acute radiation sickness with those infected by the RNA viruses, which concluded that certain DNA damage-related proteins were expressed more highly in both types of patient, consistent with the overall hypothesis.

 The final part describes an attempt to calculate the effects of viral genomes on the availability of SRSF1 in cells. The authors assume that the Kd is 0.8 µM. The Kd term they use is taken from assays with just the second RRM domain, but the value with both RRM domains is around 0.2 µM (Anczukow *et al.* (2015)) ^1^. The native protein is, of course, affected by its RS domain and therefore the state of phosphorylation. The authors also assume that there is no free protein, but this is not supported by any arguments. Taking the Kd to be 0.2 µM, the cellular concentration of the protein to be 3.6 µM (Hein *et al*. (2015)) ^2^ and the concentration of sites to be as described by the authors, this seems reasonable. However, and this is a significant caveat, the protein is not distributed evenly throughout the cell: it is largely nuclear and, within the nucleus, may be sequestered in speckles. Thus, the concentration in the cytoplasm, where it would encounter the viral RNA, might be much lower and thus affect the authors’ argument. The authors’ model involves competition between the nascent transcripts (nuclear) and the viral RNA (cytoplasmic) for SRSF1 binding, and any difference in the local concentrations and proportions of sites bound would undermine the model. However, none of the values required are known accurately and so, again, for the purpose of developing a model it is reasonable to make these simplifying assumptions.

 Overall, this is an interesting piece of work that makes the best use of the limited data available to support a model that proposes new and plausible routes by which RNA viruses could cause widespread apoptosis. It would be improved by a more rigorous discussion of the assumptions made, as noted above.

Is the work clearly and accurately presented and does it cite the current literature?

Yes

If applicable, is the statistical analysis and its interpretation appropriate?

Yes

Are all the source data underlying the results available to ensure full reproducibility?

Yes

Is the study design appropriate and is the work technically sound?

Yes

Are the conclusions drawn adequately supported by the results?

Yes

Are sufficient details of methods and analysis provided to allow replication by others?

Yes

Reviewer Expertise:

RNA splicing.

I confirm that I have read this submission and believe that I have an appropriate level of expertise to confirm that it is of an acceptable scientific standard.

## References

[1] : SRSF1-Regulated Alternative Splicing in Breast Cancer. *Mol Cell*.2015;60(1) : 10.1016/j.molcel.2015.09.005 105-17 10.1016/j.molcel.2015.09.005 26431027PMC4597910

[2] : A human interactome in three quantitative dimensions organized by stoichiometries and abundances. *Cell*.2015;163(3) : 10.1016/j.cell.2015.09.053 712-23 10.1016/j.cell.2015.09.053 26496610

# Response

**Published:** 2020-12-11

**Authors:** Peter Rogan

**Affiliations:** University of Western Ontario, Canada

*It was assumed, but has not yet been tested, that the sites elected are the stronger binding sites. It is not clear whether this has any biological relevance, i.e., how bound lifetimes (affinity) vary across all the sequences in the model and whether the threshold chosen is likely to reflect the real behaviour of the proteins.*

 Response: The IWMs derived in this manuscript may not yet been verified in the laboratory, however we have verified binding sites by such approaches previously (Vyhlidal, Rogan et al. *J Biol Chem*. 2004. 279:46779-86). The information theory framework used to derive them has been well validated and the relationships between information content and binding affinity have been rigorously proven (Schneider. *J Theor Biol.* 1997; 189:427-41). While the models used in this study were recently derived, other information theory-based RNA-protein binding site models have been utilized in hundreds of published studies, some involving the verification of phenotypes of mutations that alter splice sites, and others applied to transcription factor binding site recognition (Rogan et al. *Hum Mutat.* 1998; **12**: 153–171; Rogan et al. *Pharmacogenetics.* 2003; **13**: 207–218; Caminsky et al. F1000Res. 2014; 3:282; Lu et al. Nucl. Ac. Res. 45: e27, 2017). Our assumptions regarding binding site strength are well founded both theoretically and experimentally for many proteins. Quantification of the predicted strengths of these binding sites made by these models is reasonable and may likely reflect actual protein binding events.

*The Kd term they use is taken from assays with just the second RRM domain, but the value with both RRM domains is around 0.2 µM (Anczukow et al. (2015).* *The authors also assume that there is no free protein, but this is not supported by any arguments.*

 Response: We are grateful to the reviewer for pointing out the updated dissociation constant of SRSF1 (Anczukow et al. 2015; Ref. 54), based on assays that included both RRM domains. We had inadvertently overlooked this study. The K _d_ value used in the initial version of our paper (0.8µM) was based on an earlier publication from the same group (Cléry et al. *Proc Natl Acad Sci U S A.* 2013; 110(30):E2802–2811).  The new K _d_ value altered Scatchard analysis presented in Figure 6. The number of doublings for replicated virus was significantly increased, which reduced the discrepancy with the number of viral genomes required to compete with host transcriptome biding sites in A549 cell lines. In this revision, we have recomputed all values based on K _d_ from Anczukow et al. (2015) and have updated the main text, Figure 6 and Section 1 Tables 9A and 9B (extended data; Ref. 39).

 We previously did not include a justification for our assumption that [L] = 0 (no free protein) in our derivation of the Scatchard plot. The proposed mechanism relies on the likelihood that these RBPs are largely sequestered by binding to viral sequences, so that their effective concentration in the nucleus is inadequate to prevent R-loops (thus, [L] ≈ 0). SARS-CoV-2 replication is highly efficient and rapid, leading to levels constituting up to 60% of the total cellular RNA (Blanco-Melo et al. 2020; Ref. 55). Viral replication produces an excess of viral binding sites that will perturb the equilibrium between bound and free RBPs (Le Chatelier’s Principle) and drive binding of free RBPs and reduce the pool of free RBPs. The degree of viral replication that depletes nuclear RBP concentrations to a point at which the abundance of these factors becomes insufficient to prevent R-loop formation is not known.

 To clarify our assumption that all RBPs are bound to viral (or host), we have added the following to the Results:

      “We assumed no free RNA binding protein (that the vast majority of SRSF1 is bound to either host or viral binding sites) as the concentration of free RBPs is likely to be low due to sequestration of RBPs by the excess of viral sequences present in infected cells (~60% of all RNA ^55 ^)”

 and to the Methods:

      “Upon infection and viral replication, it is assumed there is no free RNA binding protein (all RBP is assumed to be bound to either viral or host RNA).”

*However, and this is a significant caveat, the protein is not distributed evenly throughout the cell: it is largely nuclear and, within the nucleus, may be sequestered in speckles. Thus, the concentration in the cytoplasm, where it would encounter the viral RNA, might be much lower and thus affect the authors’ argument. The authors’ model involves competition between the nascent transcripts (nuclear) and the viral RNA (cytoplasmic) for SRSF1 binding, and any difference in the local concentrations and proportions of sites bound would undermine the model.*

 Response: The reviewer has commented that the difference in local RBP concentrations between the cytoplasm and the nucleus would have an impact on the amount of RBP (such as SRSF1) that could possibly be sequestered in the cytoplasm by viral RNA. We do not state that we assume that these RBPs are uniformly distributed within the cell. We suggest that viral RNA binds to newly synthesized RBPs that have been translated in the cytoplasm, which could result in such an imbalance by limiting their nuclear entry (last paragraph of the “Proposed molecular pathogenetic mechanism of RNA-viral infection” section). It was also illustrated in panels 3 and 4 of the infographic of our proposed mechanism (Ref. 28; ( http://doi.org/10.6084/m9.figshare.12718799.v2).

# Peer review: Reviewer response for version 1

**DOI:** 10.5256/f1000research.28014.r73887

**Published:** 2020-11-24

**Authors:** Gregory Fonseca

**Affiliations:** 1 Division of Experimental Medicine, Department of Medicine, McGill University, Montreal, Canada

In this study, the authors present evidence that R-loops are associated with RNA binding protein binding sites and this may lead to DNA damage associated apoptosis. The authors define IWMs for RNA binding proteins, SRSF1 and RNPS1 based on previously published, high quality data. They then show the occurrence and quality of these IWMs in viral genomes including the relative stability of these IWMs across evolution. The authors then compared the quality and relative quantity of these IWMs in the human transcriptome. They predict the number of viral RNA particles necessary to squelch RNA binding proteins from the human genome.

 Overall, this is an extremely interesting paper with a very exciting hypothesis. The paper is well written and well organized and makes use of available datasets very well.

 A few notes.

- It should be mentioned whether IWM discovery was compared to background to understand if IWNs are found above random chance.
- If you randomly curate IWMs from 5000 sites of Rep1 or bin 5000 sites do the results compare to Rep2?
- What would the predicted likelihood of IWMs changing by chance compare to observed?
- Is there a correlation of SRSF and RNPS1 sites in the mRNA and gene expression at basal and during infection?

Is the work clearly and accurately presented and does it cite the current literature?

Yes

If applicable, is the statistical analysis and its interpretation appropriate?

I cannot comment. A qualified statistician is required.

Are all the source data underlying the results available to ensure full reproducibility?

Yes

Is the study design appropriate and is the work technically sound?

Yes

Are the conclusions drawn adequately supported by the results?

Yes

Are sufficient details of methods and analysis provided to allow replication by others?

Yes

Reviewer Expertise:

Bioinformatics, transcriptomics, genomics. Lung disease.

I confirm that I have read this submission and believe that I have an appropriate level of expertise to confirm that it is of an acceptable scientific standard, however I have significant reservations, as outlined above.

# Response

**Published:** 2020-12-11

**Authors:** Peter Rogan

**Affiliations:** University of Western Ontario, Canada

*1. It should be mentioned whether IWM discovery was compared to background to understand if IWNs are found above random chance.*

 Response:

 We derived IWMs from (mock) control binding studies for SRSF1 and RNPS1. Sequences obtained for 3 negative controls were generated in the same dataset that was used to build IWMs for RNPS1 (E-MTAB-4215; control datasets ‘ERR1201436’, ‘ERR1201437’ and ‘ERR1201438’). These were samples containing a GFP-tag (lacking the RNPS1 fusion protein for pulldown). IWMs were also derived from two SRSF1 control datasets (‘ENCFF241ORF’ and ‘ENCFF773PUP’; positive and negative strands from mock input sample). From these datasets, we derived IWMs of length 6nt from the top 5,000 peaks from each dataset with Maskminent using the same parameters (see Methods).

 The motifs of the newly derived control IWMs were compared to the true SRSF1 and RNPS1 models using STAMP software (described in Methods). The e-value obtained from STAMP is the number of hits expected against a database of the same size (i.e. 5,000 sequences containing random sequences of the same length). The comparison is based on the log likelihood ratio of the e-values of the IWM motifs derived from the RBP bound sequences relative to the sequences obtained from the mock control, which is a modified LOD score. The LOD scores for the RNPS1 model ranged from 3.8 to 6.1 depending on which control IWM was compared, and for SRSF1, these scores ranged from 4.4 to 8.8. We therefore conclude that the motifs obtained for these protein binding sites are *significantly* more robust (and different from a random set of sequences of the same size and composition generated from control samples). Please note that this analysis has been incorporated into the manuscript (the fourth paragraph of the Results and in the second and fourth paragraphs of the Methods).

*2. If you randomly curate IWMs from 5000 sites of Rep1 or bin 5000 sites do the results compare to Rep2?*

 Response:

 In this study, the two SRSF1 IWMs utilized were derived from two separate replicates from publicly available eCLIP data. The first model “SRSF1 Replicate 1” was based on the 50,000 largest eCLIP peaks from the ‘ENCFF179SCM’ replicate, while “SRSF1 Replicate 2” was based on the top 5,000 peaks in the ‘ENCFF184TBM’ replicate. Despite being derived from a far smaller dataset, the models were computed to be quite similar and non-random by STAMP analysis (e-score: 7.4e-10).

 While the SRSF1 “Replicate 1” and “Replicate 2” models were those which were selected to be used in the study, IWMs for SRSF1 were derived utilizing a series of different conditions (i.e. number of peaks, number of Monte Carlo cycles, etc.), however discussion of these additional derived SRSF1 models was not included in the final manuscript. Most commonly, models derived under these varying conditions were similar to that of the final models.

 On occasion, the method used here has been reported to identify binding motifs of other factors whose binding site sequences are in close proximity with the factor being crosslinked, as well as IWMs with a noise motifs that can resemble repetitive sequences (Lu et al. Nucleic Acids Res. 2017 Mar 17;45(5):e27). We carefully evaluated each IWM before it was utilized in any downstream analyses. For example, while the SRSF1 model derived from 10,000 peaks from the ‘replicate 1’ dataset is highly similar to that of the 50,000 replicate 1 peak model (as well as those models derived from replicate 2 data), the 5,000 replicate 1 peak model contained a slight variation of the primary motif, reporting instead an unexpected “G[G/C]AG” sub-motif.  The pairwise IWM e-values from comparison of SRSF1 Replicates 1 and 2 are: “SRSF1 Replicate 2” (self comparison): 3.9e-11;  “SRSF1 Replicate 1” (top e-CLIP 50,000 peaks): 7.4e-10;  “SRSF1 Replicate 1” (top 10,000 peaks): 3.6e-09;  and “SRSF1 Replicate 1” (top 5,000 peaks: 5.0e-03.  In general, however, modifying the number of binding sites from which models are derived generally leads to IWMs with highly similar binding motifs.

*3. What would the predicted likelihood of IWMs changing by chance compare to observed?*

 Response:

 See response to Question 1.

*4. Is there a correlation of SRSF and RNPS1 sites in the mRNA and gene expression at basal and during infection?*

 Response:

 The expression of basal SRSF1 and RNPS1 may be significantly altered in an infected cell. Blanco-Melo et al. (Ref. 55) performed differential gene expression analysis of A549 immortalized cell lines, comparing infection with influenza A- or SARS-CoV-2 with controls and ranking genes by fold change p-values adjusted for multiple testing. Both SRSF1 and RNPS1 exhibited statistically significant lowered expression in SARS-CoV-2 infected A549 cell lines (73% [p=3.7e-16] and 47% [p=3.5e-89] of controls, respectively). Their expression was also significant decreased in SARS-CoV-2 infected Calu-3 cell lines (74% for SRSF1 and 75% for RNPS1). Expression of SRSF1 and RNPS1 in A549 cells infected with respiratory syncytial virus was also significantly reduced (73% for both). No significant differences were evident in A549 infected with either SARS-CoV-1, MERS nor influenza A. Expression of SRSF1 and RNPS1 is not only altered by viral infection, but the extent of these changes is related to the specific infectious pathogen.

 We have added a statement to the manuscript acknowledging that the gene expression datasets utilized in this study were from uninfected cells which may differ in infected cells (new text bolded):

        “These are derived from the number of SRSF1 and RNPS1 sites expressed in either a single A549 cell or a type II pneumocyte **(cells were not infected; note that infection would be expected to alter the expression profile, which could affect expressed binding site estimates)**.”

# Peer review: Reviewer response for version 1

**DOI:** 10.5256/f1000research.28014.r73885

**Published:** 2020-11-18

**Authors:** Mansi Srivastava

**Affiliations:** 1 Indiana University School of Medicine, Indianapolis, IN, USA

This study provides mechanistic insights into the RNA viral infection that triggers unrepaired sites of chromosomal breakage, causing apoptosis and consequentially, high-titer viral release. The hypothesis suggests that the viral genome binds RNA binding proteins of the host thus, preventing their essential post-transcriptional activities.

 In the result section that describes the human transcriptome analysis of RNA binding sites, the authors evaluate the frequency of RBP binding in human transcriptomes to relate the relative abundance of these proteins bound to viral RNAs compared to their normal reservoir in host nuclear RNA of infected cells. To support their observation, authors should include a discussion on the impact of other RNA binding proteins that may bind/regulate the same site on the viral genome. 

 Authors discuss that the viral genome may have an advantage in the competition for binding to RBPs relative to nuclear transcripts, due to the proximity of the viral genome to nascent RBPs in the cytoplasm, which may limit transport and impede their import into the nucleus. However, the authors should also mention RBPs that shuttle between the nucleus and cytoplasm dynamically and thus account for binding to the nascent transcripts at the basal level.

Is the work clearly and accurately presented and does it cite the current literature?

Yes

If applicable, is the statistical analysis and its interpretation appropriate?

Yes

Are all the source data underlying the results available to ensure full reproducibility?

Yes

Is the study design appropriate and is the work technically sound?

Yes

Are the conclusions drawn adequately supported by the results?

Yes

Are sufficient details of methods and analysis provided to allow replication by others?

Yes

Reviewer Expertise:

Genomics and Systems Biology of RNA regulatory processes.

I confirm that I have read this submission and believe that I have an appropriate level of expertise to confirm that it is of an acceptable scientific standard.

# Response

**Published:** 2020-12-11

**Authors:** Peter Rogan

**Affiliations:** University of Western Ontario, Canada

Thank you for reviewing our manuscript. Newly synthesized RBPs imported into the nucleus and bound to nascent transcripts do not necessarily have an impact on R-loop formation. Our study put a strong focus on SRSF1 and RNPS1, which have been documented to be antagonistic to R-loop formation. We have not investigated other RNA binding proteins that are known to stabilize nascent transcripts, such as the THO complex, *PCF11*, and the exoribonucleases *EXOSC3* and *EXOSC10* (Santos-Pereira & Aguilera. *Nat Rev Genet*. 2015. 16: 583-597). The CLIP data required to analyze RNA for binding sites is not currently available for many of these RBPs.

 Ribosomal RNA (rRNA) constitutes the most abundant RNA in the cytoplasm (indeed, the cell), and would likely be the most likely to interact with SRSF1 and RNPS1. Ribosomal proteins interacting with rRNA-scaffold would likely represent the most abundant competitor to viral RNAs in infected cells. rRNA interactions have a structural basis (e.g. bulged duplexes, hairpin loops) that explains their affinity for ribosomal proteins. This contrasts with the sequence-specific binding by SRSF1 and RNPS1 and other RBPs containing one or more RRM domains (Ciriello et al. BMC Bioinformatics. 2010; 11(Suppl 1): S41). The method we describe does not detect or quantify the type of structural interactions seen in rRNA and ribosomal proteins. The present approach cannot determine whether viral RNAs could bind to ribosomal proteins.

# Peer review: Reviewer response for version 1

**DOI:** 10.5256/f1000research.28014.r73888

**Published:** 2020-11-13

**Authors:** Maurizio Romano

**Affiliations:** 1 Department of Life Sciences, University of Trieste, Trieste, Italy

In the present manuscript, starting from the observation that riboviruses can cause fatal pulmonary in some infected patients, the Authors propose the interesting hypothesis that depletion of host RNA binding proteins from nuclear RNA bound to replicating viral sequences might be part of the mechanism that trigger apoptosis and viral release.

 Information theory-based analysis was used to test interactions between RBPs and individual sequences in different virus, since expression of viral sequences might sequester RBPs (the study is focused on SRSF1 and RNPS1). It is proposed a correlation RBPs depletion / destabilization of R-loops / chromosomal breakage.

 The stoichiometry of inhibition of RBPs in host nuclear RNA has been estimated by counting competing binding sites in replicating viral genomes and host RNA.

 It is concluded that the RNA virally-induced apoptosis could lead to release significant quantities of membrane-associated virions and cause the fatal pulmonary.

 Although functional analyses might be helpful to strengthen the validity of the proposed mechanism, all the steps and conclusions of the study are sufficiently clear to support the hypothesis.

 The Discussion might be shortened by taking out aspects that are not directly related to the proposed theory.

Is the work clearly and accurately presented and does it cite the current literature?

Yes

If applicable, is the statistical analysis and its interpretation appropriate?

Yes

Are all the source data underlying the results available to ensure full reproducibility?

Yes

Is the study design appropriate and is the work technically sound?

Yes

Are the conclusions drawn adequately supported by the results?

Yes

Are sufficient details of methods and analysis provided to allow replication by others?

Yes

Reviewer Expertise:

Splicing; Neuroscience.

I confirm that I have read this submission and believe that I have an appropriate level of expertise to confirm that it is of an acceptable scientific standard.

# Response

**Published:** 2020-12-11

**Authors:** Peter Rogan

**Affiliations:** University of Western Ontario, Canada

Thank you for reviewing our manuscript. We concur that the Discussion section of the manuscript is lengthy. We have therefore removed the paragraph beginning with ““The immune system appears to be a witness, rather than a direct participant …”, as it is only tangentially related from the mechanism being proposed.
